# A comparison of traditional plant knowledge between Daman people and Tibetans in Gyirong River Valley, Tibet, China

**DOI:** 10.1186/s13002-023-00583-7

**Published:** 2023-05-05

**Authors:** Chang-An Guo, Xiaoyong Ding, Huabin Hu, Yu Zhang, Ciren Bianba, Ba Bian, Yuhua Wang

**Affiliations:** 1 Department of Economic Plants and Biotechnology, Yunnan Key Laboratory for Wild Plant Resources, Kunming Institute of Botany, Chinese Academy of Sciences, 132# Lanhei Road, Heilongtan, Kunming, 650201 Yunnan China; 2 University of Chinese Academy of Sciences, Beijing, China; 3 National Centre for Borderland Ethnic Studies in Southwest China, Yunnan University, Kunming, 650091 China; 4 CAS Key Laboratory of Tropical Plant Resources and Sustainable Use, Xishuangbanna Tropical Botanical Garden, Chinese Academy of Sciences, Mengla, 666303 Yunnan China; 5 Daman Village, Gyirong Town, Gyirong County, Shigatse City, 857000 Tibet Autonomous Region China

**Keywords:** Himalayas, Ethnobotany, Biodiversity hotspots, Daman people, Traditional knowledge

## Abstract

**Background:**

By comparing the differences in plant use between various cultures or regions, we can gain a better understanding of traditional knowledge of plant use among different groups, which may lead to a more objective understanding. Even though the Tibetan and Daman people live in the same ecosystem in Gyirong town, China, their cultural backgrounds and livelihoods differ. Therefore, the objective of this study is to document the traditional knowledge of plant use among the Daman people and compare it with the local Tibetan knowledge of plant use. By doing so, we aim to explore the relationship between plant selection and use and the cultural backgrounds of different groups.

**Methods:**

During fieldwork, ethnobotanical data were collected using various methods including free listings, key informant interviews, and semi-structured interviews. To quantify the importance of plant species in the Daman people’s culture, the culture importance index, informant consensus factor index, and The Index of Agreement on Species consensus (IASc) were used. In addition, we cited previous ethnobotanical survey data from the Tibetan in Gyirong. To more comprehensively compare the differences in plant use between the Daman and Tibetan, this study constructed a knowledge network to compare the knowledge differences between the two groups.

**Results:**

In this study, traditional knowledge was collected from 32 Daman informants, resulting in a total of 68 species belonging to 39 families mentioned by Daman people and 111 species mentioned by Tibetans. Of these, 58 plants were used by both populations. The plants were classified into 3 categories and 28 subcategories, with 22 identical classes in both groups. The majority of use categories showed a high degree of sharing in both groups, and the Tibetan people had more plant use categories than the Daman people. Five plants with IASc value > 0.5 were identified in both groups: *Rhododendron anthopogon* D. Don, *Artemisia japonica* Thunb., *Juniperus indica* Bertol., *Gastrodia elata* Blume, and *Rheum australe* D. Don. The analysis of the knowledge network revealed a 66% overlap between the knowledge of the Daman and the knowledge of the Tibetans. Additionally, the plant knowledge of Tibetan people was found to be richer and more complex than that of the Daman people. However, the Daman people possess 30 unique knowledge items.

**Conclusions:**

From the perspective of plant use, the history of the Daman people's discrete migration on the border between China and Nepal allows them to retain their own knowledge of plant use. The status quo of joining Chinese nationality and settling in Gyirong town allows them to gradually integrate into the local Tibetan society. In summary, despite living in the same ecosystem and biodiversity background, the plant utilization of the Daman people and Tibetans still shows significant differences, which are due to their different cultural backgrounds and social status.

**Supplementary Information:**

The online version contains supplementary material available at 10.1186/s13002-023-00583-7.

## Background

In previous comparative ethnobotanical studies, the focus was on two aspects. On the one hand, the differences in plant use by the same cultural group in different regions were explored, aiming to investigate human adaptation to different ecological environments [1–7]. On the other hand, the differences in plant use by different cultural groups or the same group at different temporal scales in the same region were examined, aiming to explore the influence of cultural change on plant use [8–12]. Identity and cultural customs were important topics in these studies. Through comparison, a better understanding of the differences in plant use between different cultures or regions can be achieved, which can provide a more objective understanding of a group's traditional knowledge of plant use.

In Gyirong, China, there are two cultural groups living there: the Tibetan and the Daman people. The Tibetan people are one of the 56 ethnic groups recognized by the Chinese government, and they have been engaged in farming and animal husbandry in Gyirong for a long time. In addition, although the Daman people currently identify as Tibetan, their cultural background differs greatly from the local Tibetans. The Daman people were once a “Diasporas” group: "Diasporas" refers to special immigrant groups that have dispersed across the world during different historical periods. These groups leave their homes for various reasons and often live between multiple spaces and cultures [13]. Due to their multiple identities, they are able to connect with two or more societies at the same time [14]. Historical examples of "Diasporas" include the Jews and the Hmong, who have endured hardships but remained resilient [15]. Despite living in foreign lands, members of these ethnic groups maintain a sense of ethnic identity and cultural characteristics [15, 16]. Along with globalization and transnational migration, “Diasporas” have become a significant focus of anthropological and ethnici studies [17–19]. Many "Diasporas" also live along the long border of China, and their ethnic and national identities impact how they seek survival and development under state power, as well as the long-term stability of the country's border areas [20, 21].

Unlike immigrants in the era of globalization, the Daman people have resided in the border areas between China and Nepal for over two centuries [22]. They were stateless until 2003 when they were granted Chinese nationality. From a realistic perspective, the Daman people have already achieved a stable life and settled in Gyirong as Tibetan ethnicity.

In the historical context of their prolonged wandering, the Daman people faced the predicament of survival and chose to proactively join Chinese nationality, gradually integrating into the local Tibetan society [22]. They also consciously or unconsciously adopted many local customs, languages, and cultures under the new biocultural background. For instance, they celebrate the Tibetan New Year with Tibetans and partake in traditional Tibetan foods such as butter tea. Despite integrating into the local Tibetan society, the Daman people still preserve some of their own ethnic customs, such as the "Dashai Festival," which takes place on the 8th day of the eighth month of the Tibetan calendar and involves the sacrifice of sheep blood. Moreover, they have inherited ironworking skills, which have become an important aspect of their identity [14].

Previous studies have mainly explored the national identity of the Daman people from the perspectives of anthropology and political science. This study is part of an ethnobotanical study of Gyirong, and previous research on the ethnobotany of the Gyirong Tibetan people has already been published [23]. The purpose of this study is to document the traditional knowledge of plant use among the Daman people in Gyirong town and compare it with the local Tibetan knowledge of plant use, in order to explore the relationship between plant selection and use and the cultural backgrounds of different groups.

## Materials and methods

### Study area

**Fig. 1:**
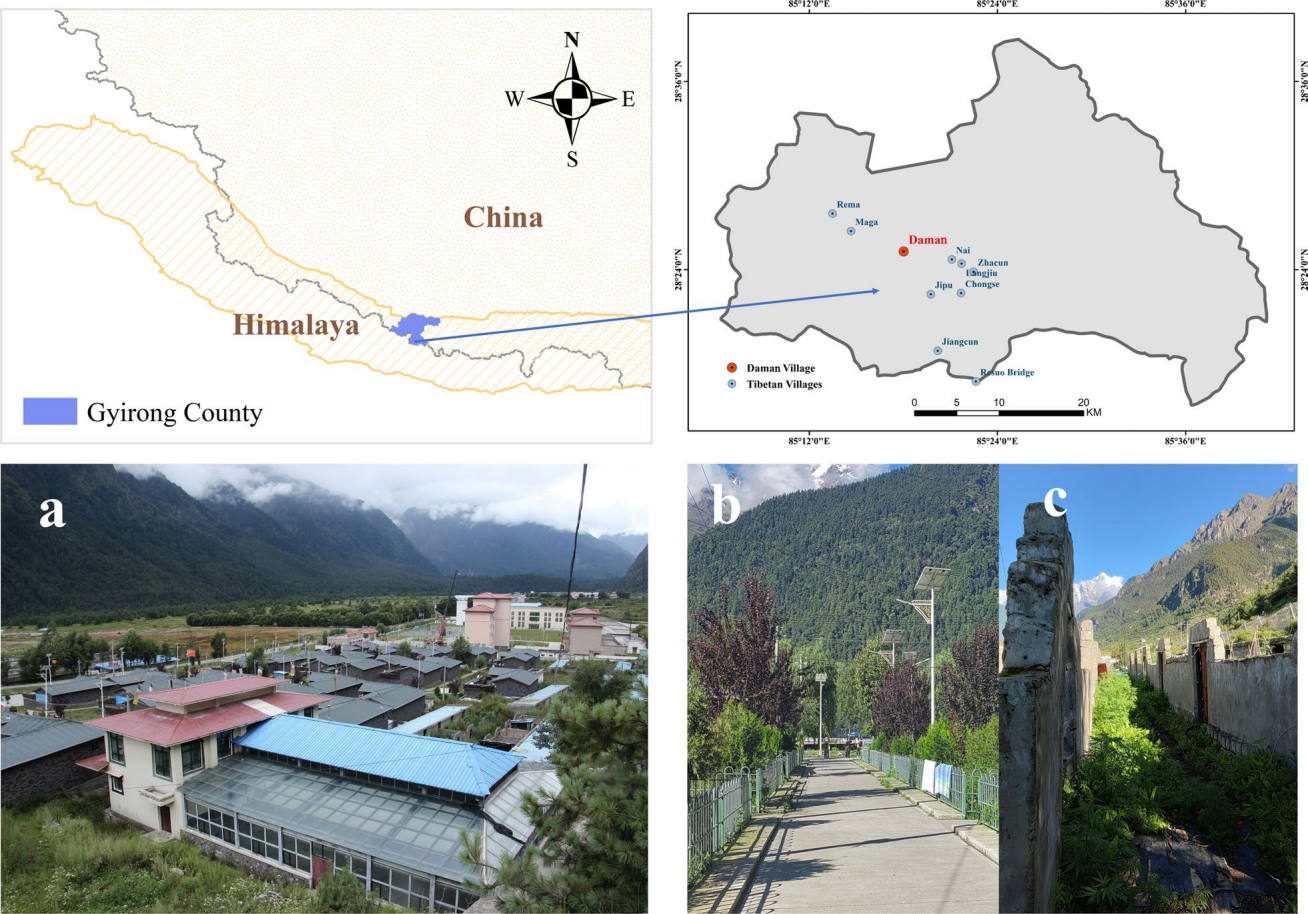
The location and environment of the Daman settlement. **a** An overall view of Daman village, **b** the road in Daman village and **c** Daman village before the earthquake

Gyirong Town is situated in the south of Gyirong County, Shigatse City, Tibet Autonomous Region, China, and is located in the core area of Mount Everest Reserve. It is bordered by Nepal to the south, and the area is characterized by an average temperature of 10–13 °C and dominated by mountain coniferous forest and mixed coniferous and broad-leaved forest vegetation types. Known as the "back garden of the Himalayas," Gyirong Town attracts tourists from around the world [24–27]. Daman Village, with a population of 207 people in 57 households, is situated about 30 km from the China-Nepal border (Fig. 1). In 2015, a magnitude-8.1 earthquake hit Nepal and caused damage to villages in Gyirong, including Daman Village. After the earthquake, the Chinese government initiated the renovation of Daman Village in Gyirong Town (Fig. 1).

### Daman people

The Daman people, who are descendants of Nepalese ancestry, have distinct physical features such as blue eyes, long eyelashes, dark brown skin, and mostly curly hairstyles [14]. These features contrast with the traditional Tibetan physical traits of brown eyes, tawny skin, and straight hair. Due to their nomadic lifestyle, the Daman people have been living along the Sino-Nepalese border for a long time [28]. With no arable land or means of production, life for the Daman people has been extremely difficult. Their language is Tibetan.

The settlement of the Daman people, also known as "Oriental Gypsy," is located in the northwest of Gyirong Town at coordinates 85.29 east longitude and 28.41 north latitude. With an average altitude of 2880 m, it was established with government funding in 2011 and is officially known as Daman New Village. Due to the lack of arable land and means of production, the Daman people rely on part-time work, farmer subsidies, and traditional handicraft cooperatives for income. There is no agriculture or animal husbandry in the village. Additionally, the village is rich in under-forest resources, and the collection of wild economic plants has become an important source of income for the Daman people [22].

### Tibetans in Gyirong

The Tibetans are one of the 56 ethnic groups in China, and their distribution is divided into three regions based on dialects: Ü-Tsang, Kham, and Amdo. The Tibetans living in Gyirong Town belong to the Ü-Tsang dialect area. The local Tibetans have a rich tradition of knowledge, including handicrafts and medicinal plant knowledge, among others [26, 27, 29].

### Field survey and data collection

In September 2019 and September 2021, our ethnobotany fieldwork was conducted in Gyirong. First, field study permission was obtained from the local community committee and government authority. We explained our purpose to local governments and requested assistance from them. All our fieldwork was conducted with informed consent. The snowball sampling method was used to select the key informants. Other informants were selected by the randomized household interview method. In addition, we attempted to train Daman guides to conduct ethnobotanical semi-structured interviews. In total, traditional knowledge was collected from 32 Daman (Table 1). The data on Tibetan ethnicity is cited from a previous ethnobotanical survey [23]. It is important to emphasize that we trained two young Daman people to conduct the fieldwork with us. The proportion of Daman informants in the study is 15% of the entire Daman population, while the Tibetan informants account for 5% of the local Tibetan population. Based on our fieldwork experience, the number of reported knowledge by the informants increases as the number of informants increases, but when the number of informants reaches a certain level, the amount of new knowledge reported no longer increases. Therefore, the selection of Daman and Tibetan informants is based on this principle, which ensures that the knowledge obtained can represent a certain group. In the selection of informants, this study strives to ensure a distribution of informants of both genders and different age groups (Table 1).

**Table 1:** Characteristics of informants

Characteristics	Tibetan		Daman	
*Gender*				
Female	61	51%	15	47%
Male	59	49%	17	53%
*Age*				
Below 20	5	5%	5	16%
20–29	12	12%	8	25%
30–39	19	19%	5	16%
40–49	27	27%	6	19%
50–59	26	26%	3	9%
60–69	21	21%	4	13%
70–79	7	7%	1	3%
Above 80	3	3%	0	0%

Ethnobotanical knowledge was collected by semistructured face to face interviews. Because many Tibetans in the study area cannot speak Mandarin fluently, the field work was performed with the assistance of local guides who were employed with the help of local community leaders. All interviews were conducted in the Tibetan language, which was translated into Mandarin by local guides. All field studies were conducted with the consent of informants. The use reports of each informant for the plants were recorded. According to the commonly used 5W + 1H (What, Where, When, Who, Why, How) principle in ethnobotany, this study designed the following questions for semistructured interviews:

1. Would you mind listing some wild plants you have used?
2. How to use this plant?
3. Which plant parts were used, roots, stems, leaves or other parts?
4. Why do you use this species?
5. What time do you collect this plant?

The questions were designed to collect data on the (1) vernacular name of the plants, (2) category of use, (3) parts used, (4) methods for preparation and administration, (5) characteristics of the plant material (dried or fresh) and (6) collection time.

The specimens were collected from the field of survey with the help of the key informants and all materials are labelled with numbers and names. Photographs of each plant were taken. All specimens were kept in the herbarium of Kunming Institute of Botany (KUN). The Flora of China was used as a reference to identify the plants [30] and The Plants of the World Online was used to ensure the Latin name of the plants [31].

### Data analysis

We adopted the use report (UR), cultural important index (CII) as ethnobotanical indice. All information about the use of local plants was organized into a “use report” list consisting of three parts: informant, used plant and used category [32, 33].

The cultural important index (CII) was the sum of the proportion of informants that mentioned each of the use categories for a given species [34]. This index is used to quantitatively evaluate the importance of a certain plant to Yadong Tibetans from the perspective of comprehensive value. In other words, CI represents the diversity of plant uses and the degree of recognition of information sources for each use category. The calculation formula is as follows:

$$\user2{CII } = \user2{ }\mathop \sum \limits_{{\user2{U } = \user2{ u}1}}^{{{\varvec{uNC}}}} \mathop \sum \limits_{{\user2{i } = \user2{ i}1}}^{{{\varvec{iN}}}} \frac{{{\varvec{URui}}}}{{\varvec{N}}}$$

NC was the total number of use categories and N was the total number of informants. CII ranges between 0 and the number of all use categories. A higher CII value indicated the multiple uses of a species and a higher degree of recognition.

The informant consensus factor index (FIC) was developed by Robert T. Trotter [35]. FIC was used to evaluate the degree of consensus among the population about how to treat a particular disease. The calculation formula is as follows:

$${\varvec{FIC}} \, = \frac{{{\varvec{Nur}} - {\varvec{Nt}}}}{{{\varvec{Nur}} - 1}}$$

where Nur is the number of use reports from the informants for a particular disease and Nt is the total number of plant species used to treat the disease. The FIC values range between 0 and 1. A higher FIC means that different herbalists have a higher consensus on the plant species used to treat certain diseases.

The Index of Agreement on Species consensus (IASc) was used to identify the proportion of culturally important species in each groups [36]. It can be assumed that it is a quantitative measure that reflects the degree of agreement or consensus among members of the group regarding their knowledge of different plant species. Overall, the research is attempting to investigate the relationship between plant knowledge and group consensus, and is using the IASc value as a way to operationalize and measure consensus within the group.

$${\varvec{IASc}} \, = \frac{{{\varvec{Pu}} \times \left( {{\varvec{ns}} - {\varvec{nu}}} \right)}}{{{\varvec{Pt}} \times \left( {{\varvec{ns}} - 1} \right)}}$$

where Pu represents the number of participants who reported a use, and Pt equals the total number of participants interviewed about the species, ns is the number of use reports of a given species mentioned by all the participants and nu is the number of use types attributed to that species. IASc values vary between 0 and 1, with 0 representing no agreement sand 1 total agreement. In this paper, we determined the proportion of plant species with an IASc value > 0.5; this value was chosen as an arbitrary cutoff point for culturally important species following Vandebroek [36].

### Indigenous knowledge network

We define knowledge as the combination of plant species and their uses. Previous comparisons focused solely on plant species and uses, but the differences in plant knowledge between two populations can be more effectively compared using the concept of knowledge networks [37]. Knowledge networks are graphs that depict the relationships between different types of plant knowledge. We drew our knowledge network using the "ggalluvial" package in R 4.2.2.

## Results

### Wild useful plant diversity and frequently utilized species

The Daman people mentioned a total of 68 species and subspecies from 39 families, with the majority belonging to Rosaceae (11), Compositae (7), and Polygonaceae (4). Herbaceous plant species were the most commonly used by the Daman people (42 species, 60.9%), followed by trees (17, 24.6%) and shrubs (10, 14.5%). *Pinus wallichiana* A.B.Jacks (UR = 32, CII = 1.000) and *Rhododendron anthopogon* D. Don (32, 1.000) were the most frequently used plants, followed by *Polygonatum cirrhifolium* (Wall.) Royle (29, 0.906), *Artemisia japonica* Thunb. (26, 0.813), and *Gastrodia elata* Blume (26, 0.813) (Table 2). In comparison, Tibetans in Gyirong mentioned a total of 111 species and subspecies from 39 families [23].

**Table 2:** List of plants used by Daman people in Gyirong

Local name(s)	Botanical family	Botanical taxon	Voucher	Parts used	Local use (no. of urs)	CII	IASc
jia1-duo1-suo3-wa1	Adoxaceae	*Viburnum nervosum* D. Don	QTB-JL-102	Fruits	Food:fruit (14), eaten raw	0.438	0.438
niu1-cei1-ma1	Amaranthaceae	*Chenopodium album* L	QTB-JL-42	Leaves	Food:vegetable (18), stir-fried	0.563	0.563
guo1-ba1	Amaryllidaceae	*Allium chrysanthum* Regel	QTB-JL-21	Bulbs	Food:seasoning (1), cooked with other Food;vegetable (2), stir-fried	0.063	0.000
zen1-bu1;ri3-guo3	Amaryllidaceae	*Allium przewalskianum* Regel	QTP-EBT-3200	Whole plants	Food:seasoning (6), cooked with other food;vegetable (18), stir-fried	0.750	0.628
guo1-nie1	Apiaceae	*Carum carvi* L	QTB-JL-63	Leaves	Food:vegetable (1), stir-fried	0.031	–
ba1-ji1	Apocynaceae	*Cynanchum auriculatum* Royle ex Wight	QTB-JL-79	Fruits	Food:fruit (6), eaten raw	0.188	0.188
dong1-ma1-ei1-ma1	Araceae	*Arisaema tortuosum* (Wall.) Schott	QTB-JL-77	Tubers	Food:starche (2), ground, fermented, and then cooked	0.063	0.063
jia1-cei1-ma1	Araliaceae	*Aralia* sp.	QTB-JPG-10	Leaves	food:vegetable (3), stir-fried	0.094	0.094
san1-jing1	Araliaceae	*Panax pseudoginseng* Wall	QTP-EBT-3084	Roots	Medicine: tonic (7), decoction	0.219	0.219
ra1-ma1-xia3-jia1	Asparagaceae	*Polygonatum sibiricum* F.Delaroche	QTB-JL-26	Roots	Food:vegetable (9), stir-fried; medicine: tonic(20), decoction	0.906	0.723
jia1-la-1-suo3-wa1; giu1-lu1	Berberidaceae	*Berberis xanthophlaea* Ahrendt	QTB-JL-27	Leaves	Medicine:diarrhea (2), decoction	0.063	0.063
da1-ge1-ba1	Betulaceae	*Betula utilis* D.Don	QTB-JL-7	Branches	Fulewood (2), burned	0.063	0.063
mang1-zhu1-cei1-ma1	Brassicaceae	*Thlaspi arvense* L	QTB-JL-35	Leaves	Food:vegetable (13), stir-fried	0.406	0.406
sei1-ma1	Cannabaceae	*Cannabis sativa* L	QTB-JL-78	Barks	Tool (3), used to make rope	0.094	0.094
bang1-bu4	Caprifoliaceae	*Nardostachys jatamansi* (D.Don) DC	QTB-JL-123	Roots	Medicine:cough (1), decoction; ritual use (11), burned	0.375	0.313
bang1-ma1	Compositae	*Artemisia calophylla* Pamp	QTB-JL-50	Aerial parts	Ritual use (4), burned; medicine: rheumatism (2), decoction	0.188	0.150
sang1-kang1-ba1	Compositae	*Artemisia japonica* Thunb	QTB-JL-59	Aerial parts	Ritual use (18), burned; medicine: rheumatic arthritis (8), decoction	0.813	0.660
bang1-ma1-ge1-dong1	Compositae	*Artemisia younghusbandii* J. R. Drumm. ex Pamp	QTB-JL-49	Aerial parts	Medicine: fever (1), decoction	0.031	–
di1-di1-li1	Compositae	*Crepis elongata* Babc	QTB-JL-98	Roots	Medicine:digestion (5) gynaecopathia (6), decoction	0.344	0.309
gang3-la1-mei3-duo1	Compositae	*Saussurea tridactyla* Sch.Bip. ex Hook.f	QTB-JL-66	Whole plants	Economic (1), be sold; medicine:arthritis (12), decoction	0.406	0.344
si1-li1-mei3-duo3	Compositae	*Senecio raphanifolius* Wall. ex DC	QTP-EBT-3066	Whole plants	Medicine: typhia(5), decoction	0.156	0.156
se1-ji1-mei3-duo3	Compositae	*Taraxacum sikkimense* Hand.-Mazz	QTB-JL-110	Whole plants	Medicine:endocrine (2), decoction	0.063	0.063
ca1-lu1	Coriariaceae	*Coriaria terminalis* Hemsl	QTP-EBT-3005	Fruits	Food:fruit (3), eaten raw	0.094	0.094
suo3-la1-ma3-bu4	Crassulaceae	*Rhodiola himalensis* (D. Don) S.H. Fu	QTB-JL-124	Roots	Medicine:hypertension (2), decoction	0.063	0.063
si1-lu1-mei3-duo3	Crassulaceae	*Sedum multicaule* Wall. ex Lindl	QTB-JL-94	Aerial parts	Medicine:injuries (3), apply to the affected area	0.094	0.094
ruo1-ruo1	Cucurbitaceae	*Herpetospermum pedunculosum* (Ser.) C.B. Clarke	QTB-JL-22	Seeds	Medicine:cold(2), decoction	0.063	0.063
ma1-ma1-dong3-cei1	Cucurbitaceae	*Solena heterophylla* Lour	QTB-JL-80	Fruits	Food:fruit(4), eaten raw	0.125	0.125
xiu1-bai1	Cupressaceae	*Juniperus indica* Bertol	QTB-JL-57	Branches	Ritual use(20), burned	0.625	0.625
xiu1-bo1	Cupressaceae	*Juniperus tibetica* Kom	QTB-JL-64	Branches	Ritual use(11), burned	0.344	0.344
da1	Dennstaedtiaceae	*Pteridium aquilinum* var. *latiusculum* (Desv.) Underw. ex A. Heller	QTB-JL-10	Leaves	Food:vegetable(15), stir-fried	0.469	0.469
zha1-lu1	Elaeagnaceae	*Elaeagnus umbellata* Thunb	QTB-JL-18	Fruits	Food:fruit (14), eaten raw	0.438	0.438
da1-ru1	Elaeagnaceae	*Hippophae salicifolia* D.Don	QTB-JL-16	Fruits;branches	food:fruit (3), eaten raw; seasoning (2), cooked with other food; fuelwood (1), burned	0.188	0.075
ba1-lu1	Ericaceae	*Rhododendron anthopogon* D. Don	QTB-JL-115	Branches	Ritual use (30), burned; food: beverage (2), boiled with water	1.000	0.907
mei1-dang1	Ericaceae	*Rhododendron arboreum* Sm	QTB-JL-30	Branches	Fuelwood (16), burned	0.500	0.500
tu1-tu1-le4-du3-ba4	Euphorbiaceae	*Euphorbia micractina* Boiss	QTB-JL-85	Whole plants	Medicine: poison (9), decoction	0.281	0.281
bei1-luo1	Fagaceae	*Quercus semecarpifolia* Sm	QTB-JL-25	Branches	Fulewood (3), burned	0.094	0.094
da1-ga1	Juglandaceae	*Juglans regia* L	QTB-JL-88	Pericarp; branches	Dye (18), used to dye the fruit black; fuelwood (1), burned; food: fruit (2), eaten raw	0.656	0.534
bai1-mu1	Liliaceae	*Fritillaria cirrhosa* D. Don	QTP-EBT-3012	Bulbs	Economic (1), be sold; medicine: cough, cold (12), decoction	0.406	0.344
bo1-ruo4	Liliaceae	*Lilium nepalense* D.Don	QTB-JL-120	Bulbs	Medicine:tonic(9),decoction	0.281	0.281
jiang1-ba1-la1-mu1	Malvaceae	*Malva verticillata* L	QTB-JL-36	Roots;leaves	Medicine: digestion (4), decoction; food: vegetable (14), stir-fried	0.563	0.412
tian3-ma3	Orchidaceae	*Gastrodia elata* Blume	QTP-JPG-3292	Roots	Economic (4), be sold; medicine: tonic (22), decoction	0.813	0.660
ang1-bu1-la1-ba1	Orchidaceae	*Gymnadenia orchidis* Lindl	QTB-JL-56	Roots	Medicine: tonic (3), decoction; vegetable (1), stir-fried	0.125	0.063
wang1-ya1	Phytolaccaceae	*Phytolacca acinosa* Roxb	QTB-JL-84	Roots; leaves	Medicine: poison (2), decoction; food: vegetable (11), stir-fried	0.406	0.315
tang3-ge1-ru3-bai1;tang3-xin1	Pinaceae	*Pinus wallichiana* A.B.Jacks	QTB-JL-39	Barks; seeds; branches	Food: vegetable (11), stir-fried; fruit (2),eaten raw; fuelwood (19), burned	1.000	0.731
di1-da1	Plantaginaceae	*Neopicrorhiza scrophulariiflora* (Pennell) D.Y.Hong	QTB-JL-67	Roots	Medicine: cold (16), decoction	0.500	0.500
niu1-lu1	Poaceae	*Fargesia* sp.	QTB-JL-118	Stems	Food: vegetable (9), stir-fried	0.281	0.281
jiang1-ma1	Poaceae	Poaceae sp.	QTP-JPG-8	Aerial parts	Food: fodder (2), feed the cattle	0.063	0.063
a1-lang1-ba1-lang1	Polygonaceae	*Fallopia denticulata* (C.C.Huang) Holub	QTB-JL-122	Roots	Medicine: cold (5), decoction	0.156	0.156
qu1-zha1	Polygonaceae	*Rheum australe* D. Don	QTB-JL-3	Roots, stems	Dye (19), used to dye yellow; food: fruit (3), eaten raw	0.688	0.565
xiu1-ma1	Polygonaceae	*Rumex nepalensis* Spreng	EBT-PL-86	Whole plants	Fodder (4), feed the cattle	0.125	0.125
zen1-du1	Ranunculaceae	*Aconitum gymnandrum* Maxim	QTP-EBT-3097	Whole plants	Medicine: poison, decoction (7)	0.219	0.219
beng3-ma1	Ranunculaceae	*Aconitum Gyirongense* W.T.Wang & L.Q.Li	QTB-JPG-1	Roots	Medicine: inflammation (2), detoxification (3), decoction	0.156	0.117
bo1-ge1-da1	Rhamnaceae	*Berchemia flavescens* (Wall.) Wall. ex Brongn	QTB-JL-93	Fruits	Food: fruit (18), eaten raw	0.563	0.563
bai1-la1	Rosaceae	*Chaenomeles thibetica* T.T.Yu	QTB-JL-109	Fruits	Food: fruit (6), eaten raw	0.188	0.188
bang1-sei1	Rosaceae	*Fragaria nubicola* (Lindl. ex Hook.f.) Lacaita	QTB-JL-9	Fruits	Food: fruit (10), eaten raw	0.313	0.313
chu1-ma1	Rosaceae	*Potentilla anserina* L	QTP-EBT-3055	Roots	Food: starche (6), boiled	0.188	0.188
bu1-long1-che4-mang1	Rosaceae	*Prinsepia utilis* Royle	QTB-JL-38	Fruits	Economic (2), be sold	0.063	0.063
a1-lu1-ba1-la	Rosaceae	*Prunus holosericea* (Batal.) Kost	QTB-JL-91	Fruits	Food: fruit (2), eaten raw	0.063	0.063
a1-xiu1-kang1-bu4	Rosaceae	*Prunus mira* Koehne	QTB-JL-69	Fruits	Food: fruit (20), eaten raw	0.625	0.625
gu1-jiu1-ma1	Rosaceae	*Rosa sericea* Wall. ex Lindl	QTB-JL-17	Fruits	Food: fruit (16), Burned	0.500	0.500
nia1-lang1-sei3-bo1;nia1-nang1	Rosaceae	*Rubus aurantiacus* Focke	QTB-JL-14	Fruits	Food: fruit (14), burned	0.438	0.438
nia1-lang	Rosaceae	*Rubus austrotibetanus* T.T.Yu & L.T.Lu	QTB-JL-82	Fruits	Food: fruit (18), eaten raw; medicine: dispel the effects of alcohol (1),decoction	0.594	0.531
na1-zi1	Rosaceae	*Sorbus cuspidata* (Spach) Hedl	QTB-JL-5	Fruits	Food: fruit (12), eaten raw	0.375	0.375
ca1-le1-ba1	Rosaceae	*Sorbus ochracea* (Hand.-Mazz.) Vidl	QTB-JL-92	Branches	Tool (3), used to make axe handles; fuelwood (3), burned	0.188	0.125
ei1-ma1	Rutaceae	*Zanthoxylum bungeanum* Maxim	QTB-JL-8	Pericarp	Food: seasoning (11), cooked with other food	0.344	0.344
lang1-ma1	Salicaceae	*Salix trichocarpa* C.F. Fang	QTB-JL-47	Stems	Fuelwood (3), burned	0.094	0.094
sei1-ge1-xin1	Taxaceae	*Taxus wallichiana* Zucc	QTB-JL-31	Stems	Tool (5), the stem is used to make tools	0.156	0.156
suo3-wa1	Urticaceae	*Urtica ardens* Link	QTP-JPG-5	Leaves	Food: vegetable (11), boiled with water	0.344	0.344

The two ethnic groups mentioned a total of 129 species and subspecies, belonging to 48 families. Rosaceae was the most represented family with 15 species, followed by Compositae with 9 and Polygonaceae with 6. Among these, 58 species were commonly known by both ethnic groups. Only one of the top ten plants used by Daman and Tibetans was the same, which was *Rhododendron anthopogon* D. Don, a plant used for beverages and Tibetan incense. Of the 129 species, 18 were endemic to China, one was listed as an endangered species by the Information System of Chinese Rare and Endangered Plants (ISCREP), and four were Near-threatened and seven were Vulnerable [38]. Additional details about Tibetans can be found in the supplementary material (see Additional file 4).

### Comparison of used part of plants

**Fig. 2:**
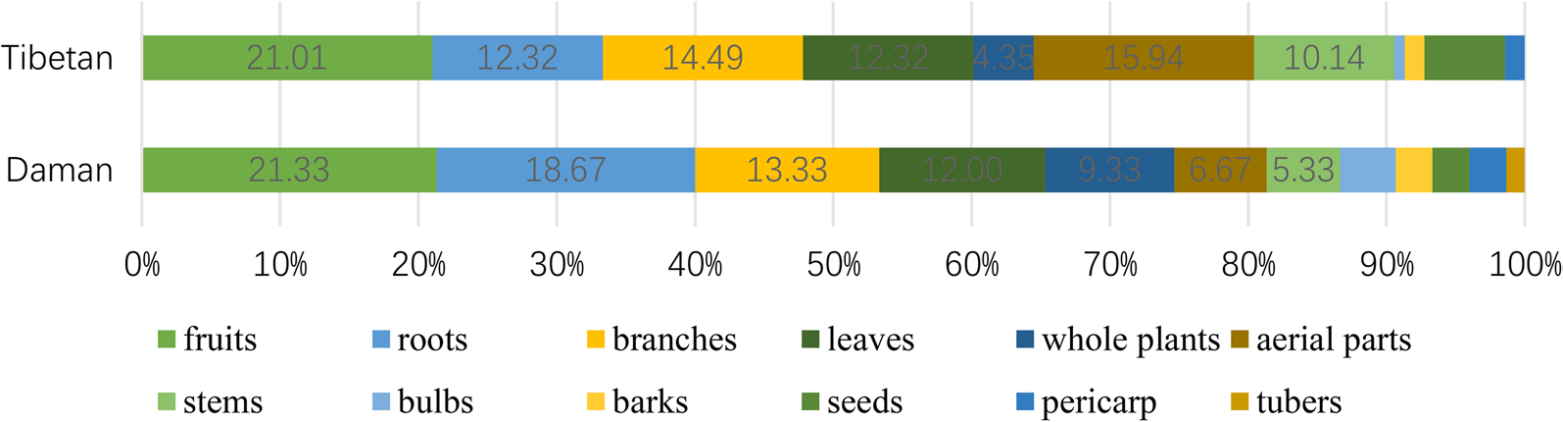
Comparison of used part of plants

Fruits were found to be the most commonly used plant part by both the Daman (21.33%) and Tibetan (21.01%) communities in Gyirong, followed by roots (18.67%) for the Daman and aerial parts (15.94%) for the Tibetans. Branches and leaves were also commonly used by both groups. Notably, the Daman people use the tubers of *Arisaema tortuosum* (Wall.), which were not used by the Tibetans (Table 2). Figure 2 provides a graphical representation of these findings.

### Comparison of ues categories

**Fig. 3:**
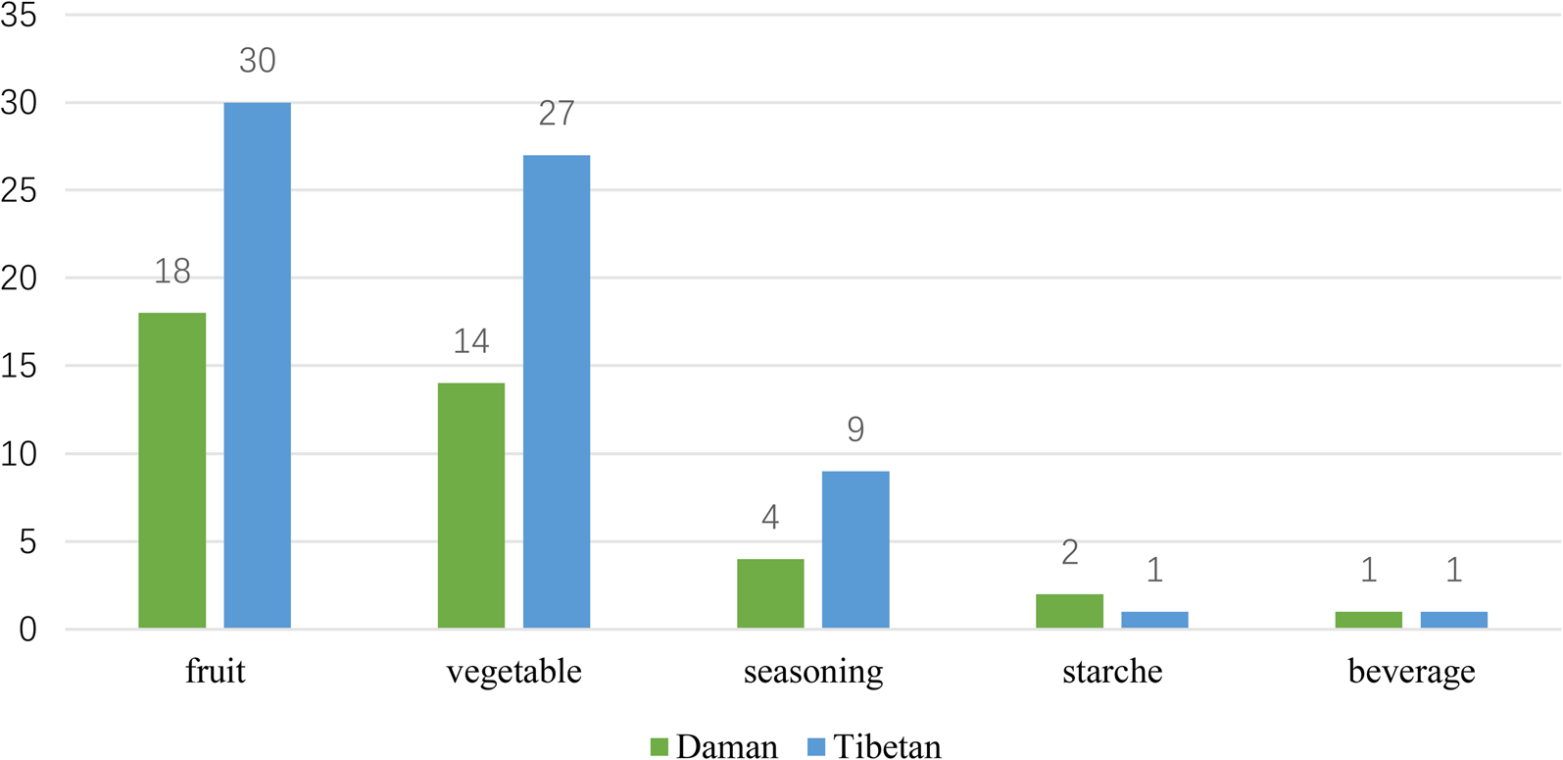
Comparison of species and quantity of edible plants

We organized all plants into 3 categories and 28 subcategories, with 22 shared subcategories between the two ethnic groups. At the main category level, both Daman and Tibetan people demonstrated similar patterns of plant use, with the majority of species being edible plants, followed by medicinal plants and other categories. However, at the subcategory level, there were significant differences. In terms of edible plants, both Daman and Tibetans primarily utilized vegetables and wild fruits, but the Tibetans employed a greater variety of edible plant species. Notably, the Daman people exhibited greater knowledge of starch plants and their uses (Fig. 3).

**Fig. 4:**
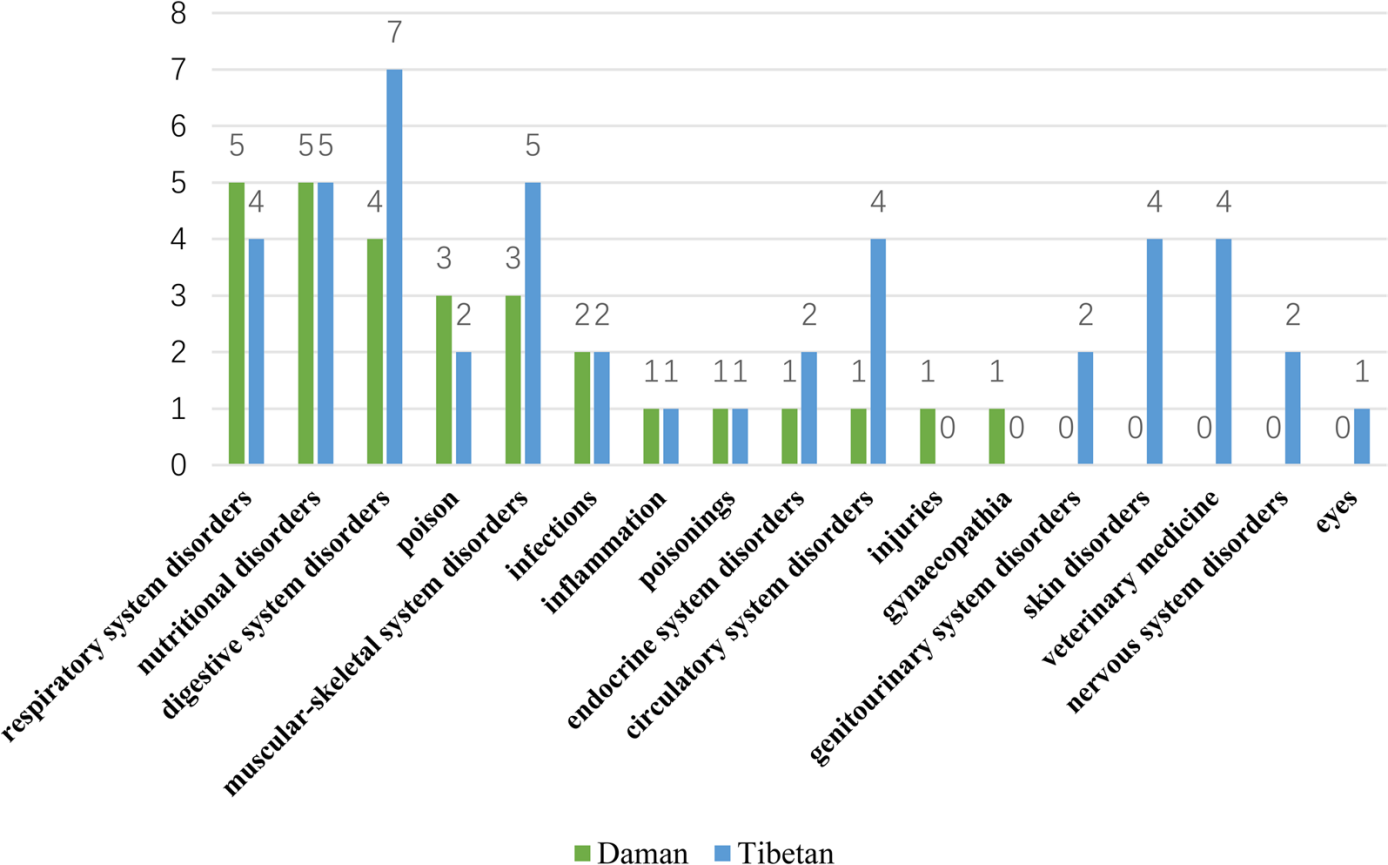
Comparison of species and quantity of medicinal plants

We categorized medicinal plants into 17 subcategories and found that the use of medicinal plants by Daman and Tibetans varied greatly, with only ten subcategories being the same. Skin disorders and veterinary medicine were the most notable subcategories, with Tibetans reporting knowledge of four plant species while the Daman people had no related knowledge (Fig. 4).

**Fig. 5:**
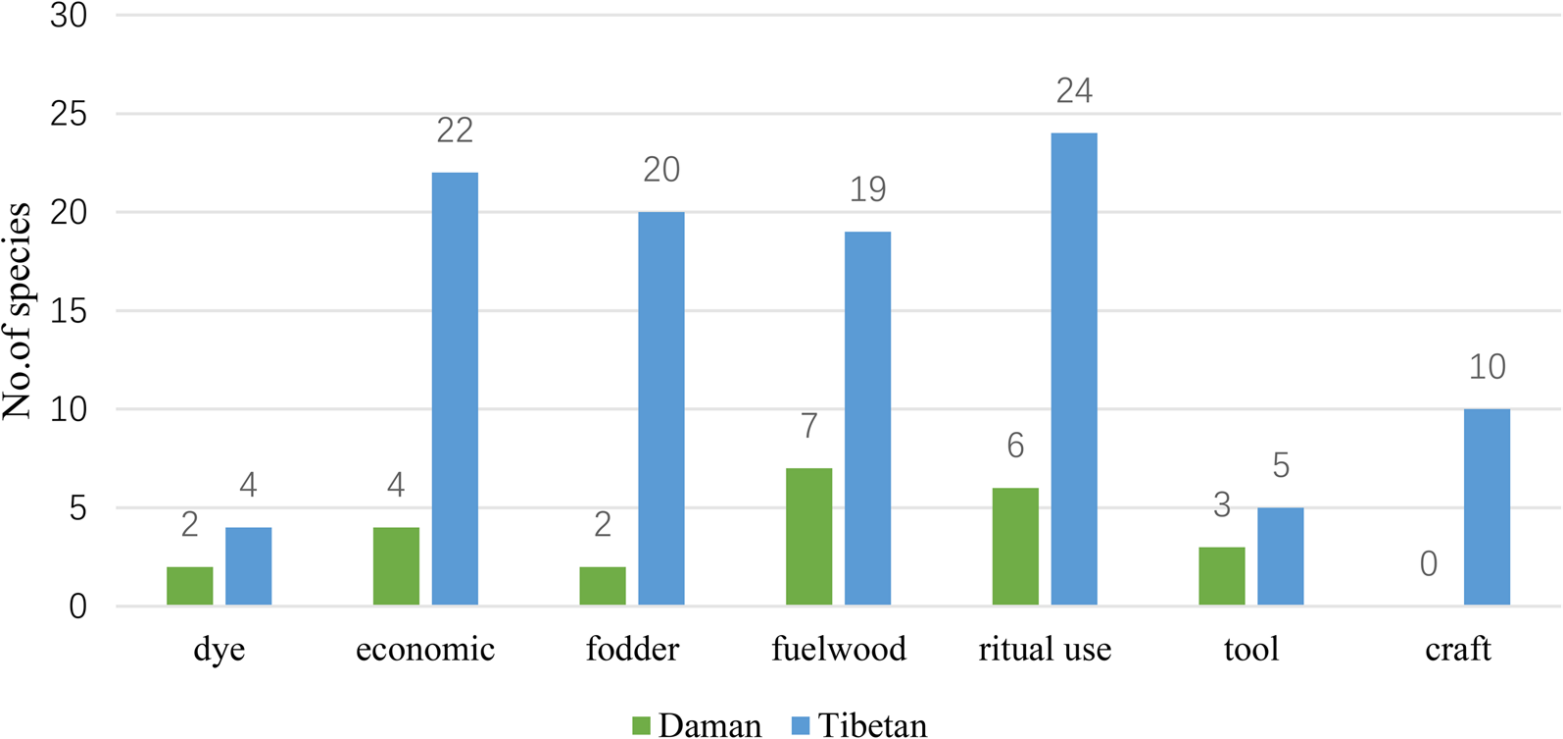
Comparison of species and quantity of other use plants

The catalog of other categories comprises 7 subcategories. The Tibetans exhibit greater richness in this aspect of plant use compared to the Daman people. Specifically, in fodder and craft, the Daman people have not reported any knowledge, while the Tibetans have demonstrated considerable knowledge (Fig. 5).

### Comparison of FIC

**Fig. 6:**
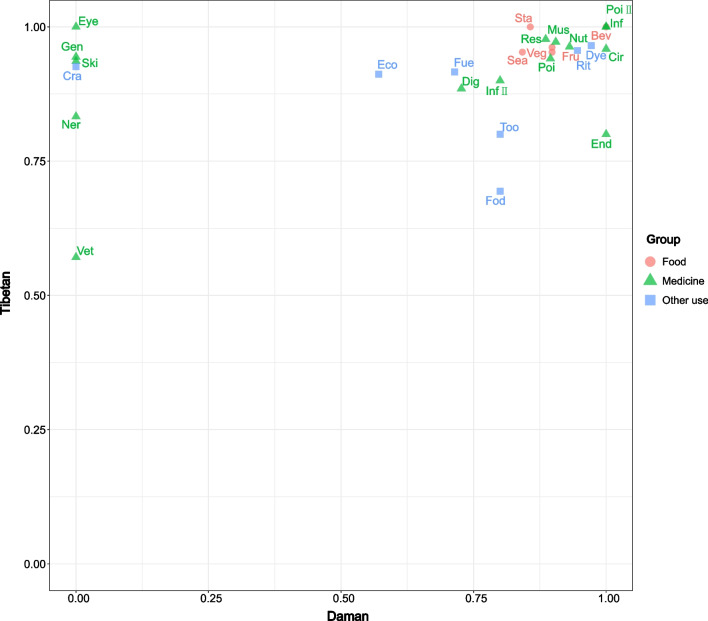
Matrix of FIC index. *Fru* fruit, *Sea* seasoning, *Veg* vegetable, *Bev* beverage, *Sta* starche, *PoiI* poison, *InfI* inflammation, *PoiII* detoxification, *InfII* infections, *dig* digestive system disorders, *Res* respiratory system disorders, *Nut* nutritional disorders, *End* endocrine system disorders, *Mus* muscular-skeletal system disorders, *Gen* genitourinary system disorders, *Ski* skin disorders, *Vet* veterinary medicine, *Ner* nervous system disorders, *Cir* circulatory system disorders, *Eye* eyes disorders, *Gyn* gynaecopathia, *Inj* injuries, *Too* tool, *Cra* craft, *Dye* dye, *Eco* economic, *Fod* fodder, *Fue* fuelwood, *Rit* ritual use

The FIC values for the 28 subcategories ranged from 0 to 1, with an average of 0.789, indicating a high degree of shared knowledge between the ethnic groups (Table 3). Both medicinal and edible plant knowledge had high FIC values, suggesting that these categories of knowledge are widely shared. Similarly, dyeing and ritual plants also had high FIC values, indicating that they are shared among the groups to a great extent. However, the use of veterinary medicinal plants had lower FIC values, suggesting that this knowledge should be particularly noted and protected (Fig. 6). Additional details can be found in the supplementary material (see Additional file 1).

**Table 3:** Use categories and FIC of plants used by Daman

Local use	Daman		FIC
	Ns	Urs	
*Edible*			
Fruit	18	167	0.8976
Seasoning	4	20	0.8421
Vegetable	14	129	0.8984
Beverage	1	2	1.0000
Starch	2	8	0.8571
*Medicine*			
Poison	3	20	0.8947
Inflammation	1	2	1.0000
Detoxification	1	3	1.0000
Infections	2	6	0.8000
Digestive system disorders	4	12	0.7273
Gynaecopathia	1	6	1.0000
Respiratory system disorders	5	36	0.8857
Nutritional disorders	5	59	0.9310
Injuries	1	3	1.0000
Endocrine system disorders	1	2	1.0000
Muscular-skeletal system disorders	3	22	0.9048
Circulatory system disorders	1	2	1.0000
*Other uses*			
Dye	2	37	0.9722
Economic	4	8	0.5714
Fodder	2	6	0.8000
Fuelwood	7	22	0.7143
Ritual use	6	94	0.9462
Tool	3	11	0.8000

### IASc matrix of two groups

**Fig. 7:**
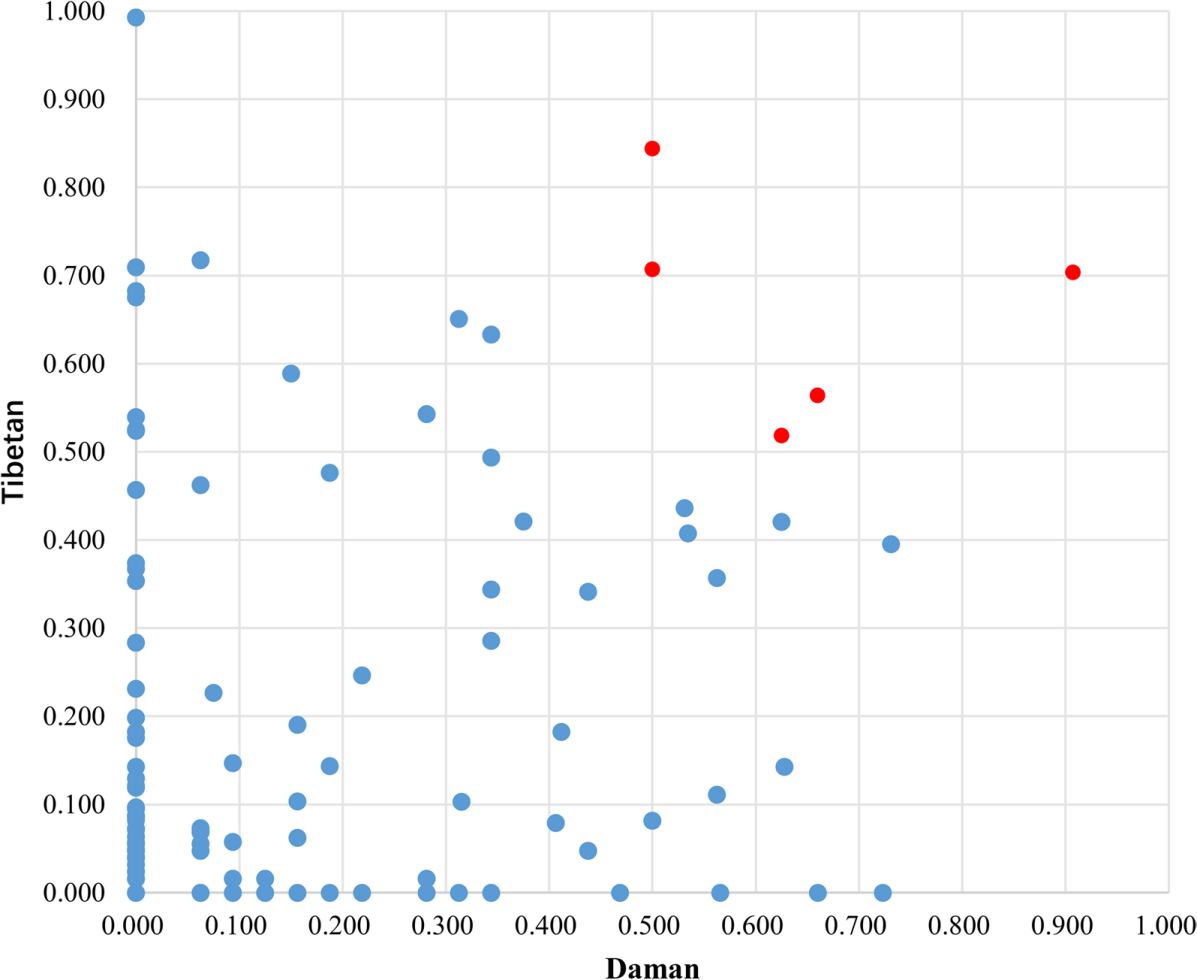
Matrix of IASc index

The aim of this study was to examine the relationship between plant knowledge and group consensus at the cultural level. To achieve this, we ranked plant species based on their IASc value and used a cutoff value of IASc > 0.5 to identify highly consented species [39]. In the Daman group, we found 13 plant species with an IASc value greater than 0.5, with *Rhododendron anthopogon* D. Don (IASc = 0.907) being the highest ranked species. Similarly, in the Tibetan group, we identified 17 plant species with an IASc value greater than 0.5, with *Allium prattii* C.H.Wright (0.993) being the highest ranked species. There were only five plant species with an IASc value greater than 0.5 in both groups, including *Rhododendron anthopogon* D. Don (IAScDaman = 0.907, IAScTibetan = 0.703), *Artemisia japonica* Thunb. (0.660, 0.564), *Juniperus indica* Bertol. (0.625, 0.518), *Gastrodia elata* Blume (0.660, 0.518), and *Rheum australe* D. Don (0.565, 0.524) (Fig. 7). Additional information is available in the supplementary material (see Additional file 2).

### Comparison of indigenous knowledge networks

**Fig. 8:**
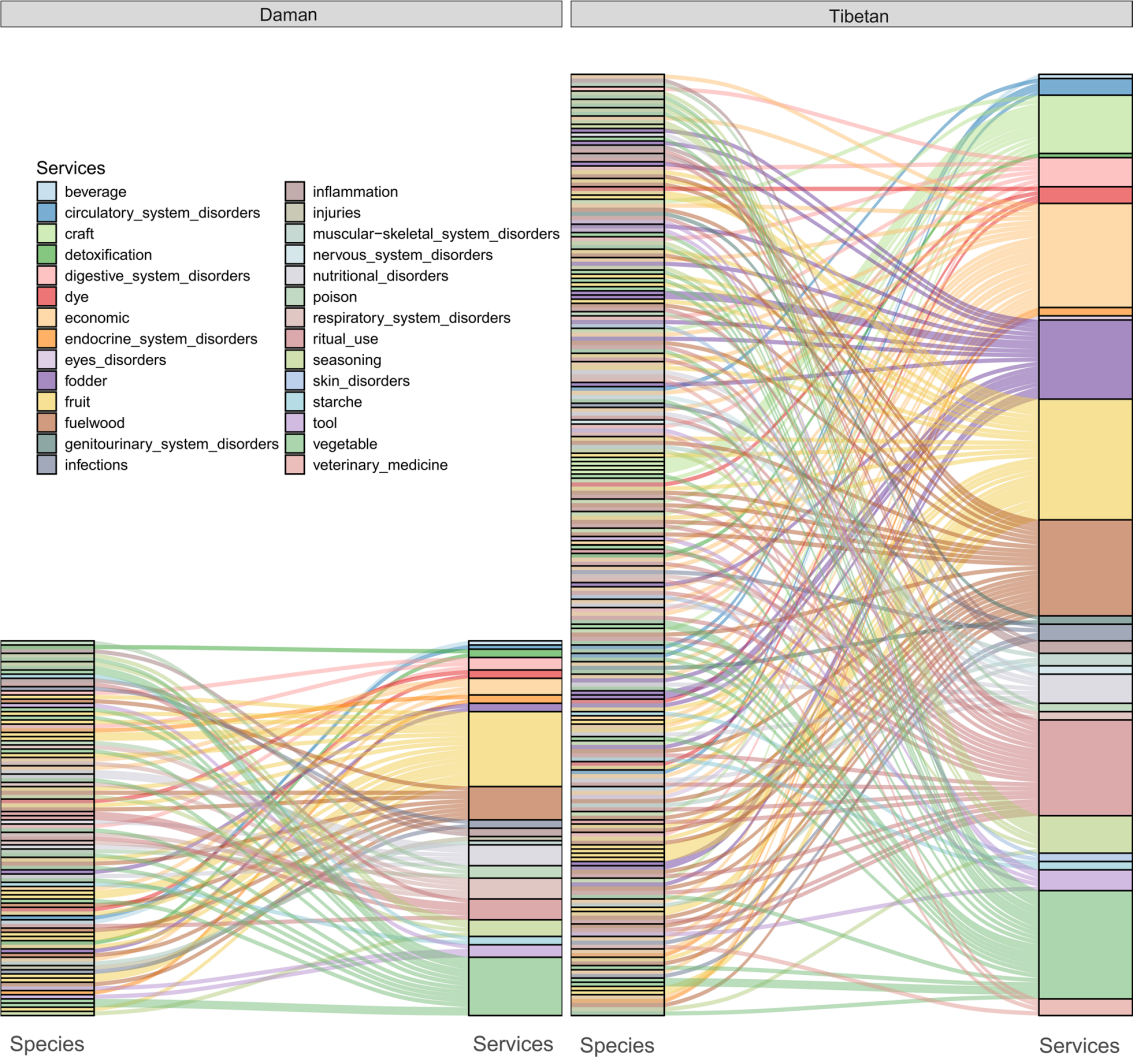
Indigenous plant knowledge networks of Daman and Tibetan

The plant knowledge network was created to provide a more visual representation of the differences in knowledge between the two cultural groups. The results indicate that the Tibetan people demonstrated a richer and more complex understanding of plants than the Daman people (Fig. 8). In terms of practical applications, as can be seen from Fig. 8, the Tibetan people possess a more extensive knowledge of economic plants, fodder, and veterinary medicine. In addition, Tibetans have more knowledge than Damans in almost all categories (Fig. 8).

**Fig. 9:**
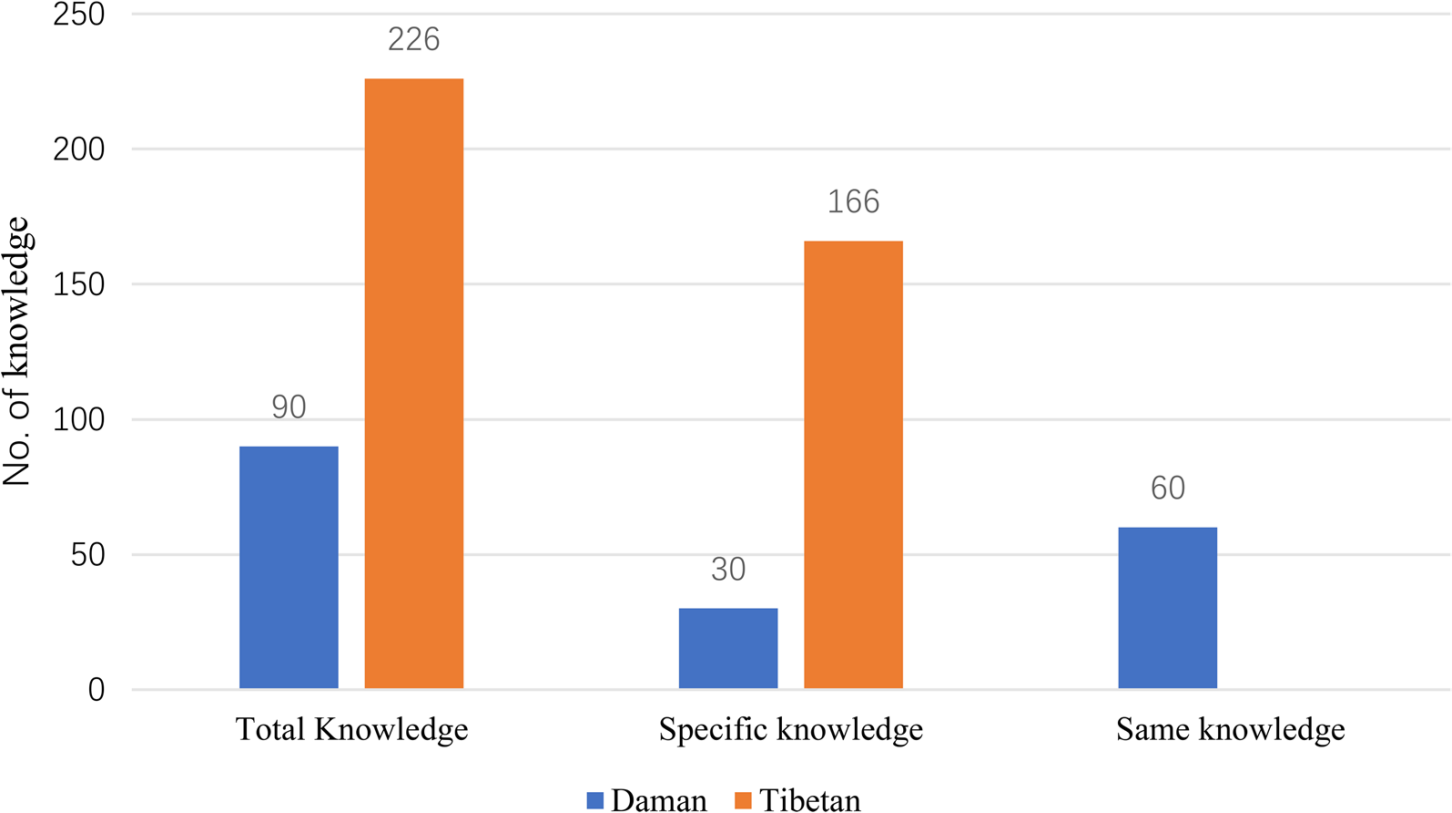
Comparison of knowledge quantity

Specifically, we found that the Daman people possessed 90 pieces of plant knowledge, while the Tibetans in Gyirong had a much larger knowledge base of 226 plant species (Fig. 9). Among the total knowledge base, there were 60 pieces of overlapping knowledge shared between the two groups. Additionally, 30 pieces of knowledge were specific to the Daman people, while 166 pieces of knowledge were specific to the Tibetans (Fig. 9). For a more detailed breakdown of these findings, please see the supplementary materials (Additional file 3).

## Discussion

### Important plant uses of Daman

Our study identified several plants that were deemed important based on their CII (cultural importance index) values. These plants were found to have a wide range of uses and were highly regarded and accepted by the local community.

*Pinus wallichiana* A.B.Jacks. is the most commonly reported wild plant in Daman village. It has a wide distribution across Bhutan, Nepal, India, and China. Our survey found that the phloem flakes of this plant were consumed directly, or processed into long strips, boiled, and used as a type of "pine bark noodles." The seeds were also eaten directly by the Daman people, while the branches and dried pine cones were highly valued as fuelwood materials. In Yadong county, the seeds are used as nuts [40]. In Nepal and India, the resin of *P. wallichiana* is used as a stimulant, stomachic, and remedy for gonorrhea. When applied externally as a plaster, it helps abscesses to suppurate. The wood is considered diaphoretic and is widely used to treat burning sensations, fainting, cough, and ulceration. Additionally, the oleoresin extracted from the wood is used to treat scorpion stings and snake bites [41, 42]. Notably, our study recorded the edible bark of *P. wallichiana* for the first time.

*Rhododendron anthopogon* D. Don is predominantly found in high altitude areas of China (southern Tibet), Bhutan, Nepal, and India. It holds significant cultural value as an important Tibetan incense plant and is widely used in various Tibetan regions, including the Tibetans of Yadong and the Lhoba of Douyu [40, 43]. The flower of *R. anthopogon* is also used as an herbal tea by people in Dolpa, Humla, and Mustang District, Nepal, and has been found to be effective in treating gastritis, common cold, indigestion, and as a diuretic [41].

*Polygonatum cirrhifolium* F. Delaroche is highly valued by the Daman people as both a wild vegetable and medicine. The young leaves of this plant are eaten as a vegetable, while its roots are used as a tonic medicine. *P. cirrhifolium* is primarily distributed in southern Tibet, Nepal, and India, and Tibetans in Yadong consider it to be an important wild economic plant [40]. The root juice of *P. cirrhifolium* is used as a tonic and taken in cases of fractures by people in Dolpa district and Mustang district, Nepal [41]. Additionally, in Manang District, Nepal, this plant is used to treat cough, fever, and to increase sexual potency [43].

*Artemisia japonica* Thunb is widely distributed in East and South Asia and is used as Tibetan incense and medicine in these areas [40, 43–46]. The Daman people use the plant to fumigate the body and treat rheumatoid arthritis by spreading it under stone slabs and setting it on fire. Additionally, it is burned as Tibetan incense by the Daman people.

*Gastrodia elata* Blume is an important economic plant for the Daman people, who collect its roots in the mountains and sell them to drug dealers. Sun-dried *G. elata* roots can fetch up to 500 yuan per 500 g, and the roots are also used as a tonic by the locals.

### Similarity of plant use between Daman people and local Tibetan people

The plant use structure and species between Daman and Tibetan communities in Gyirong exhibit high similarity. Of the 28 subcategories, 23 are the same, and 58 of the 68 plants used by the Daman people are also used by the local Tibetans. Further analysis of the FIC values revealed that the 22 identical usage categories showed a high degree of sharing between both groups (Fig. 6) [35, 47, 48]. The knowledge network analysis also showed a 66% overlap in plant knowledge between the Daman people and Tibetans. This may be due to the Daman people being scattered among Tibetans, which has led to a relatively close relationship in other aspects of social life, despite limited personal interaction [14, 22].

After analyzing the IASc of each plant, we identified five plants that occupy a critical position in both groups and belong to the first quadrant [48]. *Rhododendron anthopogon* D. Don (IASc_Daman_ = 0.907, IASc_Tibetan_ = 0.703), *Artemisia japonica* Thunb. (0.660, 0.564), and *Juniperus indica* Bertol. (0.625, 0.518) are all traditional Tibetan ritual plants used for daily incense sacrifice and are highly valued by the Daman people, playing a significant role in their lives [40, 49]. *Gastrodia elata* Blume (0.660, 0.518) is an example of a significant economic plant for the locals [50], and they collect it along with other Tibetan medicines such as *Fritillaria cirrhosa* D. Don and *Saussurea tridactyla* Sch.Bip. ex Hook.f. to improve their livelihood [51]. However, compared to the Tibetans, the Damans have limited access to the scope and quantity of these medicinal plants they can collect [52].

In summary, the plant use practices of the Daman people are highly similar to those of the local Tibetan cultural groups. The Daman people's understanding of Tibetan culture and customs has enabled them to seamlessly integrate into the local Tibetan community. This integration has facilitated better understanding and cooperation between the two groups, allowing them to coexist harmoniously.

### Differences in plant culture under the background of immigration

The perception of nature can vary between ethnic groups and is often influenced by specific cultural traditions [53]. In our study, we aimed to identify the unique cultural identity of the Daman people, while also exploring the similarities and differences in their plant use practices compared to the local Tibetans. Due to their distinct experiences and knowledge, the Daman people have developed unique methods and uses for plants that differ from those of the Tibetans. Moreover, the difference in knowledge between the Daman people and the Tibetans may be related to whether they are local indigenous people. The Daman people migrated to the Gyirong border region in the past two centuries, while the Tibetans have settled in Gyirong for thousands of years, which demonstrates the importance of indigenous knowledge [54]. The Daman people and the Tibetans have equal opportunities to access plant resources. However, the difference between the Daman people and Tibetans is that the former do not have cultivated land.

Before 2003, the Daman people were stateless and did not have access to their own land. As a result, they had to work for people in Gyirong Township or Nepal in exchange for food [14]. According to one informant, "At that time, I went to Nepal to work, and I could only exchange a handful of rice for a day of farm work." The Daman people frequently suffered from hunger and food scarcity, which compelled them to learn about edible plants in order to survive. Some of their knowledge of edible plants was passed down by local Tibetans, while other knowledge was discovered by the Daman people themselves. For instance, they recognized the importance of *Arisaema tortuosum* (Wall.) Schott as a food substitute, a plant which local Tibetans did not mention. *Sedum multicaule* Wall. ex Lindl. was also used by the Daman people to treat foot trauma, which was common due to their year-round residence in the valleys for their livelihood.

From the perspective of use categories, there still exists a cultural gap between the Daman and the Tibetans. In the area of fodder, the Daman people have very limited knowledge compared to the local Tibetans. This is due to the fact that the Daman traditionally had a small scale of cattle farming, and after the 2015 earthquake, they moved to new houses without cowsheds, which led to a further decline in their knowledge of feed plants [55].

In addition, the Daman people lack knowledge about traditional handicrafts, which is in contrast to the local Tibetans who have a wealth of botanical knowledge for making wooden bowls, with sharing practices across different villages. These differences indicate the influence of cultural background and livelihood on plant selection and use.

## Conclusion

The Daman people have a history of migrating discreetly along the China-Nepal border, which has allowed them to preserve their knowledge of plant use. The fact that they have obtained Chinese citizenship and settled in the Gyirong Daman village has resulted in a fusion between them and the Tibetan culture. In summary, while the Daman and Tibetan people live in the same ecological system with diverse species, there are still significant differences in their use of plants, which are due to their different cultural backgrounds and livelihood.

## Supplementary Information

**Additional file 1.** FIC of use categories.

**Additional file 2.** IASc of species.

**Additional file 3.** Knowledge of two groups.

**Additional file 4.** List of plants used by Tibetan people in Gyirong.

## Data Availability

Please contact the corresponding author for data requests.
