# Supplementary material for: A comparison of traditional plant knowledge between Daman people and Tibetans in Gyirong River Valley, Tibet, China
Source: J Ethnobiol Ethnomed. 2023 May 5;19:14. doi: 10.1186/s13002-023-00583-7 (PMC10163752; doi:10.1186/s13002-023-00583-7)
Supplement: Supplementary file 1 — Additional file 1. FIC of use categories. [file 13002_2023_583_MOESM1_ESM.docx]

**FIC of use categories**

| **Local use** | **Use category** | **Daman** | **Tibetan** |
| --- | --- | --- | --- |
| Food | Fru | 0.8976 | 0.9529 |
| Food | Sea | 0.8421 | 0.9535 |
| Food | Veg | 0.8984 | 0.9615 |
| Food | Bev | 1.0000 | 1.0000 |
| Food | Sta | 0.8571 | 1.0000 |
| Medicine | PoiⅠ | 0.8947 | 0.9412 |
| Medicine | InfⅠ | 1.0000 | 1.0000 |
| Medicine | PoiⅡ | 1.0000 | 1.0000 |
| Medicine | InfⅡ | 0.8000 | 0.9000 |
| Medicine | Dig | 0.7273 | 0.8846 |
| Medicine | Res | 0.8857 | 0.9774 |
| Medicine | Nut | 0.9310 | 0.9626 |
| Medicine | End | 1.0000 | 0.8000 |
| Medicine | Mus | 0.9048 | 0.9716 |
| Medicine | Gen | 0.0000 | 0.9444 |
| Medicine | Ski | 0.0000 | 0.9362 |
| Medicine | Vet | 0.0000 | 0.5714 |
| Medicine | Ner | 0.0000 | 0.8333 |
| Medicine | Cir | 1.0000 | 0.9589 |
| Medicine | Eye | 0.0000 | 1.0000 |
| Medicine | Gyn | 1.0000 | 0.0000 |
| Medicine | Inj | 1.0000 | 0.0000 |
| Other use | Too | 0.8000 | 0.8000 |
| Other use | Cra | 0.0000 | 0.9262 |
| Other use | Dye | 0.9722 | 0.9651 |
| Other use | Eco | 0.5714 | 0.9115 |
| Other use | Fod | 0.8000 | 0.6935 |
| Other use | Fue | 0.7143 | 0.9159 |
| Other use | Rit | 0.9462 | 0.9564 |
