# Supplementary material for: A comparison of traditional plant knowledge between Daman people and Tibetans in Gyirong River Valley, Tibet, China
Source: J Ethnobiol Ethnomed. 2023 May 5;19:14. doi: 10.1186/s13002-023-00583-7 (PMC10163752; doi:10.1186/s13002-023-00583-7)
Supplement: Supplementary file 2 — Additional file 2. IASc of species. [file 13002_2023_583_MOESM2_ESM.docx]

**IASc of species**

| **Plant species** | **Daman** | **Tibetan** |
| --- | --- | --- |
| *Aconitum gymnandrum* Maxim. | 0.219 | 0.247 |
| *Aconitum jilongense* W.T.Wang & L.Q.Li | 0.156 | 0.190 |
| *Allium chrysanthum* Regel | 0.000 | 0.176 |
| *Allium fasciculatum* Rendle | 0.000 | 0.097 |
| *Allium prattii* C.H.Wright | 0.000 | 0.993 |
| *Allium przewalskianum* Regel | 0.628 | 0.143 |
| *Allium wallichii* Kunth | 0.000 | 0.048 |
| *Anemone rivularis* Buch.-Ham. ex DC. | 0.000 | 0.000 |
| *Angelica sinensis* (Oliv.) Diels | 0.000 | 0.024 |
| *Aralia* sp. | 0.094 | 0.016 |
| *Aralia tibetana* G.Hoo | 0.000 | 0.040 |
| *Arisaema tortuosum* (Wall.) Schott | 0.063 | 0.000 |
| *Artemisia calophylla* Pamp. | 0.150 | 0.589 |
| *Artemisia japonica* Thunb. | 0.660 | 0.564 |
| *Artemisia younghusbandii* J. R. Drumm. ex Pamp. | 0.000 | 0.041 |
| *Avena fatua* L. | 0.000 | 0.000 |
| *Berberis angulosa* Wall. ex Hook.f. & Thomson | 0.000 | 0.049 |
| *Berberis sikkimensis* (C.K.Schneid.) Ahrendt | 0.000 | 0.024 |
| *Berberis xanthophlaea* Ahrendt | 0.063 | 0.073 |
| *Berchemia flavescens* (Wall.) Wall. ex Brongn. | 0.563 | 0.357 |
| *Betula utilis* D.Don | 0.000 | 0.130 |
| *Betula utilis* D.Don | 0.063 | 0.000 |
| *Cannabis sativa* L. | 0.094 | 0.058 |
| *Capsella bursa-pastoris* (L.) Medik. | 0.000 | 0.063 |
| *Carum carvi* L. | 0.000 | 0.354 |
| *Chaenomeles thibetica* T.T.Yu | 0.188 | 0.144 |
| *Chaerophyllum villosum* Wall. ex DC. | 0.000 | 0.016 |
| *Chenopodium album* L. | 0.563 | 0.111 |
| *Cicer microphyllum* Benth. | 0.000 | 0.016 |
| *Clematis rehderiana* Craib | 0.000 | 0.016 |
| *Coriaria terminalis* Hemsl. | 0.094 | 0.000 |
| *Crepis elongata* Babc. | 0.344 | 0.000 |
| *Cyclanthera pedata* (L.) Schrad. | 0.000 | 0.056 |
| *Cynanchum auriculatum* Royle ex Wight | 0.188 | 0.000 |
| *Delphinium kamaonense* Huth | 0.000 | 0.000 |
| *Dipsacus asper* Wall. ex C.B. Clarke | 0.000 | 0.000 |
| *Elaeagnus umbellata* Thunb. | 0.438 | 0.341 |
| *Elsholtzia fruticosa* (D.Don) Rehder | 0.000 | 0.097 |
| *Euphorbia micractina* Boiss. | 0.281 | 0.016 |
| *Fallopia denticulata* (C.C.Huang) Holub | 0.156 | 0.063 |
| *Fargesia* sp. | 0.281 | 0.543 |
| *Fragaria nubicola* (Lindl. ex Hook.f.) Lacaita | 0.313 | 0.651 |
| *Fritillaria cirrhosa* D. Don | 0.344 | 0.000 |
| *Fritillaria cirrhosa* D.Don | 0.000 | 0.710 |
| *Galinsoga parviflora* Cav. | 0.000 | 0.000 |
| *Gastrodia elata* Blume | 0.660 | 0.000 |
| *Gastrodia elata* Blume | 0.000 | 0.525 |
| Gentiana veitchiorum Hemsl. | 0.000 | 0.143 |
| *Gymnadenia orchidis* Lindl. | 0.063 | 0.717 |
| *Heracleum candicans* Wall. ex DC. | 0.000 | 0.000 |
| *Herpetospermum pedunculosum* (Ser.) C.B. Clarke | 0.063 | 0.069 |
| *Hippophae salicifolia* D.Don | 0.075 | 0.227 |
| *Holboellia angustifoli*a Wall. | 0.000 | 0.016 |
| *Impatiens bicornuta* Wall. | 0.000 | 0.087 |
| *Impatiens falcifer* Hook.f. | 0.000 | 0.087 |
| *Impatiens scabrida* DC. | 0.000 | 0.087 |
| *Impatiens sulcata* Wall. | 0.000 | 0.087 |
| *Isoetes hypsophila* Hand.-Mazz. | 0.000 | 0.198 |
| *Juglans regia* L. | 0.534 | 0.408 |
| *Juniperus indica* Bertol. | 0.625 | 0.518 |
| *Juniperus tibetica* Kom. | 0.344 | 0.344 |
| *Larix himalaica* W.C.Cheng & L.K.Fu | 0.000 | 0.073 |
| *Leontopodium souliei* Beauverd | 0.000 | 0.016 |
| *Lilium nepalense* D.Don | 0.281 | 0.000 |
| *Lonicera* sp. | 0.000 | 0.016 |
| *Malva verticillata* L. | 0.412 | 0.183 |
| *Nardostachys jatamansi* (D.Don) DC. | 0.313 | 0.000 |
| *Nardostachys jatamansi* (D.Don) DC. | 0.000 | 0.675 |
| *Neopicrorhiza scrophulariiflora* (Pennell) D.Y.Hong | 0.500 | 0.844 |
| *Nepeta densiflora* Kar. & Kir. | 0.000 | 0.024 |
| *Onosma hookeri* C.B. Clarke | 0.000 | 0.083 |
| *Panax pseudoginseng* Wall. | 0.000 | 0.374 |
| *Panax pseudoginseng* Wall. | 0.219 | 0.000 |
| *Paris polyphylla* Sm. | 0.000 | 0.283 |
| *Phytolacca acinosa* Roxb. | 0.315 | 0.103 |
| *Pinus wallichiana* A.B.Jacks. | 0.731 | 0.396 |
| *Plantago asiatica* L | 0.000 | 0.024 |
| *Plantago asiatica* L. | 0.000 | 0.040 |
| *Poaceae* sp. | 0.063 | 0.056 |
| *Polygonatum cirrhifolium* (Wall.) Royle | 0.000 | 0.232 |
| *Polygonatum sibiricum* F.Delaroche | 0.723 | 0.000 |
| *Polygonatum sibiricum* F.Delaroche | 0.000 | 0.457 |
| *Polygonum macrophyllum* D. Don | 0.000 | 0.016 |
| *Polygonum nepalense* Meisn. | 0.000 | 0.024 |
| *Polygonum tortuosum* D. Don | 0.000 | 0.084 |
| *Potentilla anserina* L. | 0.188 | 0.476 |
| *Prinsepia utilis* Royle | 0.063 | 0.071 |
| *Prunus holosericea* (Batal.) Kost. | 0.063 | 0.048 |
| *Prunus mira* Koehne | 0.625 | 0.421 |
| *Pteridium aquilinum* var. *latiusculum* (Desv.) Underw. ex A. Heller | 0.469 | 0.000 |
| *Pteridium aquilinum* var. *latiusculum* (Desv.) Underw. ex A. Heller | 0.000 | 0.683 |
| *Quercus semecarpifolia* Sm. | 0.000 | 0.368 |
| *Quercus semecarpifolia* Sm. | 0.094 | 0.000 |
| *Rheum australe* D. Don | 0.565 | 0.000 |
| *Rheum australe* D. Don | 0.000 | 0.524 |
| *Rhodiola himalensis* (D. Don) S.H. Fu | 0.063 | 0.462 |
| *Rhododendron anthopogon* D. Don | 0.907 | 0.703 |
| *Rhododendron arboreum* Sm. | 0.500 | 0.081 |
| *Rhododendron lepidotum* Wall. ex G. Don | 0.000 | 0.119 |
| *Rosa macrophylla* Lindl. | 0.000 | 0.095 |
| *Rosa sericea* Wall. ex Lindl. | 0.500 | 0.707 |
| *Rubus aurantiacus* Focke | 0.438 | 0.048 |
| *Rubus austrotibetanus* T.T.Yu & L.T.Lu | 0.531 | 0.437 |
| *Rubus biflorus* Buch.-Ham. ex Sm. | 0.000 | 0.071 |
| *Rubus niveus* Thunb. | 0.000 | 0.540 |
| *Rumex nepalensis* Spreng. | 0.125 | 0.016 |
| *Salix matsudana* Koidz. | 0.000 | 0.057 |
| *Salix trichocarpa* C.F. Fang | 0.094 | 0.147 |
| *Saussurea tridactyla* Sch.Bip. ex Hook.f. | 0.344 | 0.494 |
| *Schisandra elongata* (Blume) Baill. | 0.000 | 0.056 |
| *Sedum multicaule* Wall. ex Lindl. | 0.094 | 0.000 |
| *Senecio raphanifolius* Wall. ex DC. | 0.156 | 0.000 |
| *Solena heterophylla* Lour. | 0.125 | 0.000 |
| *Solena heterophylla* Lour. | 0.000 | 0.048 |
| *Sorbus cuspidata* (Spach) Hedl. | 0.375 | 0.421 |
| *Sorbus ochracea* (Hand.-Mazz.) J.E.Vidal | 0.000 | 0.121 |
| *Sorbus ochracea* (Hand.-Mazz.) Vidl | 0.125 | 0.000 |
| *Swertia cordata* (Wall. ex G. Don) C.B. Clarke | 0.000 | 0.073 |
| *Tamarix chinensis* Lour. | 0.000 | 0.000 |
| *Taraxacum mongolicum* Hand.-Mazz. | 0.063 | 0.000 |
| *Taraxacum sikkimense* Hand.-Mazz. | 0.000 | 0.000 |
| *Taxus wallichiana* Zucc. | 0.156 | 0.104 |
| *Thlaspi arvense* L. | 0.406 | 0.079 |
| *Trichosanthes lepiniana* (Naudin) Cogn. | 0.000 | 0.183 |
| *Urtica ardens* Link | 0.344 | 0.286 |
| Urtica urens L. | 0.000 | 0.032 |
| *Viburnum nervosum* D. Don | 0.438 | 0.048 |
| *Zanthoxylum bungeanum* Maxim. | 0.344 | 0.633 |
