# Supplementary material for: A comparison of traditional plant knowledge between Daman people and Tibetans in Gyirong River Valley, Tibet, China
Source: J Ethnobiol Ethnomed. 2023 May 5;19:14. doi: 10.1186/s13002-023-00583-7 (PMC10163752; doi:10.1186/s13002-023-00583-7)
Supplement: Supplementary file 3 — Additional file 3. Knowledge of two groups. [file 13002_2023_583_MOESM3_ESM.docx]

**Knowledge of two groups**

| **Specie** | **Service** | **Community** |
| --- | --- | --- |
| *Aconitum gymnandrum* | poison | Daman |
| *Aconitum gymnandrum* | economic | Tibetan |
| *Aconitum gymnandrum* | poison | Tibetan |
| *Aconitum gymnandrum* | inflammation | Tibetan |
| *Aconitum jilongense* | detoxification | Daman |
| *Aconitum jilongense* | inflammation | Daman |
| *Aconitum jilongense* | digestive system disorders | Tibetan |
| *Allium chrysanthum* | seasoning | Daman |
| *Allium chrysanthum* | vegetable | Daman |
| *Allium chrysanthum* | seasoning | Tibetan |
| *Allium chrysanthum* | vegetable | Tibetan |
| *Allium fasciculatum* | seasoning | Tibetan |
| *Allium fasciculatum* | vegetable | Tibetan |
| *Allium prattii* | seasoning | Tibetan |
| *Allium prattii* | vegetable | Tibetan |
| *Allium przewalskianum* | seasoning | Daman |
| *Allium przewalskianum* | vegetable | Daman |
| *Allium przewalskianum* | economic | Tibetan |
| *Allium przewalskianum* | seasoning | Tibetan |
| *Allium wallichii* | seasoning | Tibetan |
| *Anemone rivularis* | fodder | Tibetan |
| *Angelica paeoniifolia* | nutritional disorders | Tibetan |
| *Aralia sp.* | vegetable | Daman |
| *Aralia sp.* | vegetable | Tibetan |
| *Aralia tibetana* | fodder | Tibetan |
| *Arisaema tortuosum* | starche | Daman |
| *Artemisia calophylla* | inflammation | Daman |
| *Artemisia calophylla* | ritual use | Daman |
| *Artemisia calophylla* | inflammation | Tibetan |
| *Artemisia calophylla* | ritual use | Tibetan |
| *Artemisia younghusbandii* | infections | Daman |
| *Artemisia younghusbandii* | inflammation | Tibetan |
| *Artemisia younghusbandii* | ritual use | Tibetan |
| *Avena fatua* | fodder | Tibetan |
| *Berberis angulosa* | digestive system disorders | Tibetan |
| *Berberis angulosa* | fruit | Tibetan |
| *Berberis angulosa* | fuelwood | Tibetan |
| *Berberis sikkimensis* | fruit | Tibetan |
| *Berberis sikkimensis* | fuelwood | Tibetan |
| *Berberis xanthophlaea* | digestive system disorders | Daman |
| *Berberis xanthophlaea* | dye | Tibetan |
| *Berberis xanthophlaea* | fruit | Tibetan |
| *Berchemia flavescens* | fruit | Daman |
| *Berchemia flavescens* | fruit | Tibetan |
| *Betula utilis* | fuelwood | Daman |
| *Betula utilis* | craft | Tibetan |
| *Betula utilis* | genitourinary system disorders | Tibetan |
| *Betula utilis* | economic | Tibetan |
| *Betula utilis* | fuelwood | Tibetan |
| *Betula utilis* | ritual use | Tibetan |
| *Betula utilis* | tool | Tibetan |
| *Cannabis sativa* | tool | Daman |
| *Cannabis sativa* | fodder | Tibetan |
| *Cannabis sativa* | tool | Tibetan |
| *Capsella bursa-pastoris* | vegetable | Tibetan |
| *Carum carvi* | vegetable | Daman |
| *Carum carvi* | digestive system disorders | Tibetan |
| *Carum carvi* | seasoning | Tibetan |
| *Carum carvi* | vegetable | Tibetan |
| *Chaenomeles thibetica* | fruit | Daman |
| *Chaenomeles thibetica* | fruit | Tibetan |
| *Chaenomeles thibetica* | fuelwood | Tibetan |
| *Chaerophyllum villosum* | economic | Tibetan |
| *Chaerophyllum villosum* | fodder | Tibetan |
| *Chaerophyllum villosum* | seasoning | Tibetan |
| *Chenopodium album* | vegetable | Daman |
| *Chenopodium album* | vegetable | Tibetan |
| *Cicer microphyllum* | fruit | Tibetan |
| *Clematis rehderiana* | vegetable | Tibetan |
| *Coriaria terminalis* | fruit | Daman |
| *Coriaria terminalis* | fruit | Tibetan |
| *Crepis elongata* | digestive system disorders | Daman |
| *Crepis elongata* | endocrine system disorders | Daman |
| *Cyclanthera pedata* | vegetable | Tibetan |
| *Cynanchum auriculatum* | fruit | Daman |
| *Delphinium kamaonense* | fodder | Tibetan |
| *Dipsacus asper* | fodder | Tibetan |
| *Elaeagnus umbellata* | fruit | Daman |
| *Elaeagnus umbellata* | fruit | Tibetan |
| *Elsholtzia fruticosa* | fuelwood | Tibetan |
| *Elsholtzia fruticosa* | ritual use | Tibetan |
| *Euphorbia micractina* | poison | Daman |
| *Euphorbia micractina* | poison | Tibetan |
| *Fallopia denticulata* | respiratory system disorders | Daman |
| *Fallopia denticulata* | digestive system disorders | Tibetan |
| *Fallopia denticulata* | fodder | Tibetan |
| *Fallopia denticulata* | skin disorders | Tibetan |
| *Fargesia sp.* | vegetable | Daman |
| *Fargesia sp.* | craft | Tibetan |
| *Fargesia sp.* | economic | Tibetan |
| *Fargesia sp.* | fodder | Tibetan |
| *Fargesia sp.* | vegetable | Tibetan |
| *Fargesia sp.* | fuelwood | Tibetan |
| *Fargesia sp.* | ritual use | Tibetan |
| *Fragaria nubicola* | fruit | Daman |
| *Fragaria nubicola* | fruit | Tibetan |
| *Fragaria nubicola* | ritual use | Tibetan |
| *Fritillaria cirrhosa* | respiratory system disorders | Daman |
| *Fritillaria cirrhosa* | economic | Daman |
| *Fritillaria cirrhosa* | respiratory system disorders | Tibetan |
| *Fritillaria cirrhosa* | economic | Tibetan |
| *Fritillaria cirrhosa* | fruit | Tibetan |
| *Fritillaria cirrhosa* | nutritional disorders | Tibetan |
| *Fritillaria cirrhosa* | veterinary medicine | Tibetan |
| *Galinsoga parviflora* | fodder | Tibetan |
| *Gastrodia elata* | economic | Daman |
| *Gastrodia elata* | nutritional disorders | Daman |
| *Gastrodia elata* | circulatory system disorders | Tibetan |
| *Gastrodia elata* | economic | Tibetan |
| *Gastrodia elata* | nervous system disorders | Tibetan |
| *Gastrodia elata* | vegetable | Tibetan |
| *Gentiana veitchiorum* | infections | Tibetan |
| *Gymnadenia orchidi* | economic | Tibetan |
| *Gymnadenia orchidi* | ritual use | Tibetan |
| *Gymnadenia orchidi* | nutritional disorders | Tibetan |
| *Gymnadenia orchidis* | nutritional disorders | Daman |
| *Gymnadenia orchidis* | vegetable | Daman |
| *Heracleum candicans* | nervous system disorders | Tibetan |
| *Herpetospermum pedunculosum* | respiratory system disorders | Daman |
| *Herpetospermum pedunculosum* | digestive system disorders | Tibetan |
| *Herpetospermum pedunculosum* | tool | Tibetan |
| *Herpetospermum pedunculosum* | veterinary medicine | Tibetan |
| *Hippophae salicifolia* | fruit | Daman |
| *Hippophae salicifolia* | fuelwood | Daman |
| *Hippophae salicifolia* | seasoning | Daman |
| *Hippophae salicifolia* | muscular-skeletal system disorders | Tibetan |
| *Hippophae salicifolia* | fruit | Tibetan |
| *Hippophae salicifolia* | fuelwood | Tibetan |
| *Hippophae salicifolia* | seasoning | Tibetan |
| *Holboellia angustifolia* | fruit | Tibetan |
| *Impatiens bicornuta* | craft | Tibetan |
| *Impatiens falcifer* | craft | Tibetan |
| *Impatiens scabrida* | craft | Tibetan |
| *Impatiens sulcata* | craft | Tibetan |
| *Isoetes hypsophila* | vegetable | Tibetan |
| *Juglans regia* | dye | Daman |
| *Juglans regia* | fruit | Daman |
| *Juglans regia* | fuelwood | Daman |
| *Juglans regia* | craft | Tibetan |
| *Juglans regia* | dye | Tibetan |
| *Juglans regia* | fruit | Tibetan |
| *Juglans regia* | fuelwood | Tibetan |
| *Juglans regia* | ritual use | Tibetan |
| *Juniperus indica* | ritual use | Daman |
| *Juniperus indica* | craft | Tibetan |
| *Juniperus indica* | fuelwood | Tibetan |
| *Juniperus indica* | ritual use | Tibetan |
| *Juniperus tibetica* | craft | Tibetan |
| *Juniperus tibetica* | fruit | Tibetan |
| *Juniperus tibetica* | fuelwood | Tibetan |
| *Juniperus tibetica* | ritual use | Tibetan |
| *Juniperus tibetica* | ritual use | Daman |
| *Larix himalaica* | craft | Tibetan |
| *Larix himalaica* | fuelwood | Tibetan |
| *Leontopodium souliei* | tool | Tibetan |
| *Lilium nepalense* | nutritional disorders | Daman |
| *Lonicera sp.* | economic | Tibetan |
| *Malva verticillata* | digestive system disorders | Daman |
| *Malva verticillata* | vegetable | Daman |
| *Malva verticillata* | vegetable | Tibetan |
| *Myricaria rosea* | ritual use | Tibetan |
| *Nardostachys jatamansi* | respiratory system disorders | Daman |
| *Nardostachys jatamansi* | ritual use | Daman |
| *Nardostachys jatamansi* | detoxification | Tibetan |
| *Nardostachys jatamansi* | economic | Tibetan |
| *Nardostachys jatamansi* | ritual use | Tibetan |
| *Neopicrorhiza scrophulariiflora* | respiratory system disorders | Daman |
| *Neopicrorhiza scrophulariiflora* | respiratory system disorders | Tibetan |
| *Neopicrorhiza scrophulariiflora* | economic | Tibetan |
| *Neopicrorhiza scrophulariiflora* | infections | Tibetan |
| *Neopicrorhiza scrophulariiflora* | veterinary medicine | Tibetan |
| *Nepeta densiflora* | fodder | Tibetan |
| *Onosma hookeri* | economic | Tibetan |
| *Onosma hookeri* | skin disorders | Tibetan |
| *Onosma hookeri* | ritual use | Tibetan |
| *Panax pseudoginseng* | nutritional disorders | Daman |
| *Panax pseudoginseng* | economic | Tibetan |
| *Panax pseudoginseng* | nutritional disorders | Tibetan |
| *Paris polyphylla* | economic | Tibetan |
| *Paris polyphylla* | ritual use | Tibetan |
| *Paris polyphylla* | digestive system disorders | Tibetan |
| *Paris polyphylla* | vegetable | Tibetan |
| *Phytolacca acinosa* | poison | Daman |
| *Phytolacca acinosa* | vegetable | Daman |
| *Phytolacca acinosa* | vegetable | Tibetan |
| *Pinus wallichiana* | fruit | Daman |
| *Pinus wallichiana* | fuelwood | Daman |
| *Pinus wallichiana* | vegetable | Daman |
| *Pinus wallichiana* | craft | Tibetan |
| *Pinus wallichiana* | vegetable | Tibetan |
| *Pinus wallichiana* | fuelwood | Tibetan |
| *Pinus wallichiana* | ritual use | Tibetan |
| *Plantago asiatica subsp. Densiflora* | circulatory system disorders | Tibetan |
| *Plantago asiatica subsp. Densiflora* | vegetable | Tibetan |
| *Poaceae sp.* | fodder | Daman |
| *Poaceae sp.* | circulatory system disorders | Tibetan |
| *Poaceae sp.* | vegetable | Tibetan |
| *Polygonatum cirrhifolium* | economic | Tibetan |
| *Polygonatum cirrhifolium* | genitourinary system disorders | Tibetan |
| *Polygonatum cirrhifolium* | vegetable | Tibetan |
| *Polygonatum sibiricum* | nutritional disorders | Daman |
| *Polygonatum sibiricum* | vegetable | Daman |
| *Polygonatum sibiricum* | economic | Tibetan |
| *Polygonatum sibiricum* | fodder | Tibetan |
| *Polygonatum sibiricum* | nutritional disorders | Tibetan |
| *Polygonatum sibiricum* | vegetable | Tibetan |
| *Polygonum macrophyllum* | fodder | Tibetan |
| *Polygonum nepalense* | fodder | Tibetan |
| *Polygonum tortuosum* | dye | Tibetan |
| *Polygonum tortuosum* | fodder | Tibetan |
| *Polygonum tortuosum* | fruit | Tibetan |
| *Potentilla anserina* | starche | Daman |
| *Potentilla anserina* | starche | Tibetan |
| *Prinsepia utilis* | economic | Daman |
| *Prinsepia utilis* | economic | Tibetan |
| *Prunus holosericea* | fruit | Daman |
| *Prunus holosericea* | fruit | Tibetan |
| *Prunus mira* | fruit | Daman |
| *Prunus mira* | economic | Tibetan |
| *Prunus mira* | fruit | Tibetan |
| *Prunus mira* | nutritional disorders | Tibetan |
| *Pteridium aquilinum var. latiusculum* | vegetable | Daman |
| *Pteridium aquilinum var. latiusculum* | vegetable | Tibetan |
| *Quercus semecarpifolia* | fuelwood | Daman |
| *Quercus semecarpifolia* | craft | Tibetan |
| *Quercus semecarpifolia* | fodder | Tibetan |
| *Quercus semecarpifolia* | fuelwood | Tibetan |
| *Quercus semecarpifolia* | ritual use | Tibetan |
| *Quercus semecarpifolia* | starche | Tibetan |
| *Rheum australe* | dye | Daman |
| *Rheum australe* | fruit | Daman |
| *Rheum australe* | dye | Tibetan |
| *Rheum australe* | fruit | Tibetan |
| *Rhodiola himalensis* | circulatory system disorders | Daman |
| *Rhodiola himalensis* | economic | Tibetan |
| *Rhodiola himalensis* | circulatory system disorders | Tibetan |
| *Rhodiola himalensis* | ritual use | Tibetan |
| *Rhodiola himalensis* | nutritional disorders | Tibetan |
| *Rhododendron anthopogon* | beverage | Daman |
| *Rhododendron anthopogon* | ritual use | Daman |
| *Rhododendron anthopogon* | beverage | Tibetan |
| *Rhododendron anthopogon* | Muscular-skeletal system disorders | Tibetan |
| *Rhododendron anthopogon* | economic | Tibetan |
| *Rhododendron anthopogon* | eyes disorders | Tibetan |
| *Rhododendron anthopogon* | fuelwood | Tibetan |
| *Rhododendron anthopogon* | ritual use | Tibetan |
| *Rhododendron arboreum* | fuelwood | Daman |
| *Rhododendron arboreum* | craft | Tibetan |
| *Rhododendron arboreum* | fuelwood | Tibetan |
| *Rhododendron lepidotum* | ritual use | Tibetan |
| *Rosa macrophylla* | fruit | Tibetan |
| *Rosa macrophylla* | fuelwood | Tibetan |
| *Rosa sericea* | fruit | Daman |
| *Rosa sericea* | digestive system disorders | Tibetan |
| *Rosa sericea* | fruit | Tibetan |
| *Rosa sericea* | fuelwood | Tibetan |
| *Rubus aurantiacus* | fruit | Daman |
| *Rubus aurantiacus* | fruit | Tibetan |
| *Rubus austrotibetanus* | fruit | Daman |
| *Rubus austrotibetanus* | detoxification | Daman |
| *Rubus austrotibetanus* | fruit | Tibetan |
| *Rubus biflorus* | fruit | Tibetan |
| *Rubus niveus* | fruit | Tibetan |
| *Rumex nepalensis* | fodder | Daman |
| *Rumex nepalensis* | fodder | Tibetan |
| *Salix matsudana* | fodder | Tibetan |
| *Salix matsudana* | fuelwood | Tibetan |
| *Salix matsudana* | ritual use | Tibetan |
| *Salix trichocarpa* | fuelwood | Daman |
| *Salix trichocarpa* | craft | Tibetan |
| *Salix trichocarpa* | fodder | Tibetan |
| *Salix trichocarpa* | fuelwood | Tibetan |
| *Salix trichocarpa* | ritual use | Tibetan |
| *Salix trichocarpa* | vegetable | Tibetan |
| *Saussurea tridactyla* | muscular-skeletal system disorders | Daman |
| *Saussurea tridactyla* | economic | Daman |
| *Saussurea tridactyla* | muscular-skeletal system disorders | Tibetan |
| *Saussurea tridactyla* | economic | Tibetan |
| *Sedum multicaule* | injuries | Daman |
| *Senecio raphanifolius* | infections | Daman |
| *Solena heterophylla* | fruit | Daman |
| *Solena heterophylla* | fruit | Tibetan |
| *Sorbus cuspidata* | fruit | Daman |
| *Sorbus cuspidata* | fruit | Tibetan |
| *Sorbus cuspidata* | fuelwood | Tibetan |
| *Sorbus cuspidata* | ritual use | Tibetan |
| *Sorbus ochracea* | fuelwood | Daman |
| *Sorbus ochracea* | tool | Daman |
| *Sorbus ochracea* | fuelwood | Tibetan |
| *Sorbus ochracea* | ritual use | Tibetan |
| *Sorbus ochracea* | tool | Tibetan |
| *Swertia cordata* | economic | Tibetan |
| *Swertia cordata* | infections | Tibetan |
| *Swertia cordata* | veterinary medicine | Tibetan |
| *Taraxacum sikkimense* | endocrine system disorders | Daman |
| *Taraxacum sikkimense* | economic | Tibetan |
| *Taraxacum sikkimense* | endocrine system disorders | Tibetan |
| *Taxus wallichiana* | tool | Daman |
| *Taxus wallichiana* | fruit | Tibetan |
| *Taxus wallichiana* | fuelwood | Tibetan |
| *Thlaspi arvense* | vegetable | Daman |
| *Thlaspi arvense* | vegetable | Tibetan |
| *Trichosanthes lepiniana* | economic | Tibetan |
| *Trichosanthes lepiniana* | infections | Tibetan |
| *Urtica ardens* | vegetable | Daman |
| *Urtica ardens* | vegetable | Tibetan |
| *Urtica urens* | vegetable | Tibetan |
| *Viburnum cotinifolium* | fruit | Tibetan |
| *Viburnum nervosum* | fruit | Daman |
| *Viburnum nervosum* | fruit | Tibetan |
| *Zanthoxylum bungeanum* | seasoning | Daman |
| *Zanthoxylum bungeanum* | economic | Tibetan |
| *Zanthoxylum bungeanum* | endocrine system disorders | Tibetan |
| *Zanthoxylum bungeanum* | fuelwood | Tibetan |
| *Zanthoxylum bungeanum* | seasoning | Tibetan |
| *Zanthoxylum bungeanum* | vegetable | Tibetan |
