# Supplementary material for: A comparison of traditional plant knowledge between Daman people and Tibetans in Gyirong River Valley, Tibet, China
Source: J Ethnobiol Ethnomed. 2023 May 5;19:14. doi: 10.1186/s13002-023-00583-7 (PMC10163752; doi:10.1186/s13002-023-00583-7)
Supplement: Supplementary file 4 — Additional file 4. List of plants used by Tibetan people in Gyirong. [file 13002_2023_583_MOESM4_ESM.docx]

| **Botanical taxon** | **Botanical family** | **Local name(s)** | **Voucher** | **Parts used** | **Local use（No. of Urs）and Preparation** | **UR** | **CI** |
| --- | --- | --- | --- | --- | --- | --- | --- |
| *Chenopodium album* L. | Amaranthaceae | lei1; niu1; niu1-che1-ma1 | QTB-JL-42 | leaves | food: vegetable (14), used to make bun or fry. | 14 | 0.111 |
| *Allium chrysanthum* Regel | Amaryllidaceae | guo1-ba1 | QTB-JL-21 | roots | food: vegetable (20), used to make bun or fry; seasoning (7), cook it with pork and potatoes. | 27 | 0.214 |
| *Allium fasciculatum* Rendle | Amaryllidaceae | ou1-ga1 | QTP-EBT-3050 | aerial parts | food: vegetable (10), used to make bun or fry; seasoning (7), cook it with pork and potatoes. | 17 | 0.135 |
| *Allium prattii* C.H.Wright | Amaryllidaceae | ru1-ba1; ri1-guo1 | QTP-EBT-3009 | aerial parts | food: vegetable (126), used to make bun or fry; seasoning (9), cook it with pork and potatoes. | 141 | 1.119 |
| *Allium przewalskianum* Regel | Amaryllidaceae | ri1-guo1; zen1-bu1 | QTP-EBT-3200 | leaves | food: seasoning (19), as part of the dip; economic (1), be sold in stores. | 20 | 0.159 |
| *Allium wallichii* Kunth | Amaryllidaceae | ru1-guo1 | QTB-JL-55 | aerial parts | food: seasoning (6), cook it with pork and potatoes or as part of the dip. | 6 | 0.048 |
| *Angelica sinensis* (Oliv.) Diels | Apiaceae | dang1-gui1 | QTB-JL-41 | roots | medicine: tonic (3), cook it with meat. | 3 | 0.024 |
| *Carum carvi* L. | Apiaceae | guo1-nie1 | QTB-JL-63 | leaves; seeds | food: vegetable (25), the leaves are cooked with other ingredients; seasoning (35), ues seeds to make a dip or a sausage; medicine: gastralgia (5), seed soak in water | 65 | 0.516 |
| *Chaerophyllum villosum* Wall. ex DC. | Apiaceae | da1-ga1-li1 | QTB-JL-20 | leaves | economic (3), be sold in stores; food: seasoning (1), cook its leaves with meat; fodder (1): the leaves are used to feed cattle | 5 | 0.04 |
| *Heracleum candicans* Wall. ex DC. | Apiaceae | jiong4-wa1-dong1-bu4 | QTB-JL-43 | aerial parts | medicine: headache (1); soak in water. | 1 | 0.008 |
| *Aralia* sp. | Araliaceae | jia1-ra3-cei3-ma1 | QTB-JPG-10 | leaves | food: vegetable (2), cook vegetable. | 2 | 0.016 |
| *Aralia tibetana* G.Hoo | Araliaceae | dai1-ga1-ni1 | QTB-JL-46 | whole plant | fodder (5): the leaves are used to feed cattle | 5 | 0.04 |
| *Panax pseudoginseng* Wall. | Araliaceae | san1-jing1 | QTP-EBT-3084 | roots | medicine: tonic (30), soak in water; economic (25): be sold in stores. | 55 | 0.437 |
| *Polygonatum cirrhifolium* (Wall.) Royle | Asparagaceae | ra3-mu1-xia3-jia1 | QTB-JL-1 | aerial parts; roots | food: vegetable (26), cook vegetable; economic (8), be sold in store; medicine: nephropathy (11), roots soak in water or make soup. | 35 | 0.278 |
| *Polygonatum sibiricum* Redouté | Asparagaceae | rang3-ma1-xia-jia1 | QTB-JL-26 | roots; aerial parts | medicine: tonic (15), roots soak in water or make soup; food: vegetable (45), cook vegetable; economic (12), be sold in store; fodder (3): used to feed cattle. | 75 | 0.595 |
| *Artemisia calophylla* Pamp. | Asteraceae | bang1-ma1 | QTB-JL-50 | aerial parts | ritual use (4), used to burn in incense burner; medicine: rheumatism (72): used it to sweat steaming or leaves soak in water to drink. | 76 | 0.603 |
| *Artemisia japonica* Thunb. | Asteraceae | kang1-ba1 | QTB-JL-59 | aerial parts | ritual use (60), used to burn in incense burner; medicine: detoxification (23), soak in water; economic (4): be sold in store. | 87 | 0.69 |
| *Artemisia younghusbandii* J. R. Drumm. ex Pamp. | Asteraceae | sang1-kang3-ba1 | QTB-JL-49 | aerial parts | ritual use (4), used to burn in incense burner; medicine: rheumatism (4), used it to sweat steaming or leaves soak in water to drink. | 8 | 0.063 |
| *Galinsoga parviflora* Cav. | Asteraceae | cuo1-ma1 | QTP-JPG-6 | whole plants | fodder (1): used to feed cattle. | 1 | 0.008 |
| *Leontopodium souliei* Beauverd | Asteraceae | ba1-wa1 | EBT-PL-99 | leaves | tool (2), used it to start a fire. | 2 | 0.016 |
| *Saussurea tridactyla* Sch.Bip. ex Hook.f. | Asteraceae | gang3-la1-mei3-duo3 | QTB-JL-66 | whole plants | economic (36), be sold in store; medicine: arthrophlogosis (46): soaked in water. | 82 | 0.651 |
| *Taraxacum sikkimense* Hand.-Mazz. | Asteraceae | se4-ji4-mei3-duo3 | QTB-JL-110 | whole plant | medicine: endocrine (3), soaked in water; economic (2), be sold in store. | 5 | 0.04 |
| *Impatiens bicornuta* Wall. | Balsaminaceae | po1-zi1 | QTB-JL-73 | seeds | varnish (12), used to polish furniture | 12 | 0.095 |
| *Impatiens falcifer* Hook.f. | Balsaminaceae | po1-zi1 | QTB-JL-15 | seeds | varnish (14), used to polish furniture | 14 | 0.111 |
| *Impatiens scabrida* DC. | Balsaminaceae | po1-zi1 | QTB-JL-70 | seeds | varnish (13), used to polish furniture | 13 | 0.103 |
| *Impatiens sulcata* Wall. | Balsaminaceae | po1-zi1 | QTB-JL-62 | seeds | varnish (11), used to polish furniture | 11 | 0.087 |
| *Berberis angulosa* Wall. ex Hook.f. & Thomson | Berberidaceae | jiu1-bo1; jiu1-le1-bu1 | QTB-JL-113 | leaves; fruits; branches | medicine: diarrhea (1), soaked in water; food: fruit (7), raw; fuelwood (2): used to burn. | 10 | 0.079 |
| *Berberis aristata* DC. | Berberidaceae | jiu1-lu1-xin1 | QTB-JL-28 | fruits; branches | fuelwood (3), used to burn; food: fruit (2), raw. | 5 | 0.04 |
| *Berberis xanthophlaea* Ahrendt | Berberidaceae | giu1-lu1; giu1-le1-bu1; gei1-lu1-mi3-xia4 | QTB-JL-27 | barks; fruits | dyes (10), used to dye wool yellow; food: fruit (4), raw. | 14 | 0.111 |
| *Stauntonia angustifolia* (Wall.) R.Br. ex Wall. | Berberidaceae | pa1-ji1 | QTP-JPG-2 | fruits | food: fruit (2), raw. | 2 | 0.016 |
| *Betula utilis* D.Don | Betulaceae | da4-ge1-ba1 | QTB-JL-7 | burls; branches; stems | medicine: diabetes (8), soak in water; economic (2), be sold in store; fuelwood (14), used to burn; craft (7), used to make Tibetan traditional wooden bowls; ritual use (8), used to burn in incense burner; tool (6), used to make cooking utensils. | 37 | 0.294 |
| *Onosma hookeri* C.B. Clarke | Boraginaceae | guo1-mu1-mu1-zi1 | QTP-EBT-3052 | roots | medicine: hair follicle (4), soaked in canola oil and apply to the head; eczema (4); ritual use (7), used to burn in incense burner; economic (1), be sold in store. | 16 | 0.127 |
| *Capsella bursa-pastoris* (L.) Medik. | Brassicaceae | du1-yang1 | QTB-JL-34 | aerial parts | food: vegetable (8), cooked vegetable. | 8 | 0.063 |
| *Thlaspi arvense* L. | Brassicaceae | mang3-ru1 | QTB-JL-35 | leaves | food: vegetable (10), cooked vegetable | 10 | 0.079 |
| *Cannabis sativa* L. | Cannabaceae | si1-ma1 | QTB-JL-78 | barks | tool (8); fodder (4), used to feed cattle. | 12 | 0.095 |
| *Dipsacus asper* Wall. ex C.B. Clarke | Caprifoliaceae | lang1-zhu1-ma1 | QTP-EBT-3053 | aerial parts | fodder (1), used to feed cattle. | 1 | 0.008 |
| *Lonicera* sp. | Caprifoliaceae | se4-le4-qin1-mei3-duo3 | EBT-PL-42 | flowers | economic (2), be sold in store. | 2 | 0.016 |
| *Nardostachys jatamansi* (D.Don) DC. | Caprifoliaceae | bang1-bu4 | QTB-JL-123 | roots | ritual use (87), used to burn in incense burner; economic (1), be sold in store; medicine: relieving cough and asthma (4), soaked in water. | 92 | 0.73 |
| *Coriaria terminalis* Hemsl. | Coriariaceae | da1-lu1 | QTP-EBT-3005 | fruits | food: fruit (1), raw. | 1 | 0.008 |
| *Rhodiola himalensis* (D. Don) S.H. Fu | Crassulaceae | suo3-la1-ma3-bu4 | QTB-JL-124 | stems | medicine: tonic (20), hypertension (28), soaked in water; economic (19), be sold in store; ritual use (3), used to burn in incense burner. | 70 | 0.556 |
| *Cyclanthera pedata* (L.) Schrad. | Cucurbitaceae | ra3-ru1 | QTB-JPG-12 | fruits | food: vegetable (7), cooked vegetable. | 7 | 0.056 |
| *Herpetospermum pedunculosum* (Ser.) C.B. Clarke | Cucurbitaceae | sei1-lei1; sei1-lei1-mei3-duo3 | QTB-JL-22 | flowers; fruits | medicine: diarrhea (12), powder; veterinary medicine: diarrhea (3), powder. | 15 | 0.119 |
| *Solena heterophylla* Lour. | Cucurbitaceae | ma1-ma1-dong4-cei1 | QTB-JL-80 | fruits | food: fruit (6), raw. | 6 | 0.048 |
| *Trichosanthes lepiniana* (Naudin) Cogn. | Cucurbitaceae | ka1-ge1-di1 | QTB-JL-24 | seeds | medicine: fever (1), poder; economic (1), be sold in store. | 2 | 0.016 |
| *Juniperus indica* Bertol. | Cupressaceae | xiu1-bai1 | QTB-JL-57 | branches; stems | ritual use (67), used to burn in incense burner; fuelwood (7), used to burn; craft (6) | 80 | 0.635 |
| *Juniperus tibetica* Kom. | Cupressaceae | xiu1-bo1 | QTB-JL-64 | branche; stems | ritual use (38), used to burn in incense burner; fuelwood (22), used to burn; craft (20), used to make Tibetan traditional wooden bowls; food: fruit (2), raw. | 82 | 0.651 |
| *Pteridium aquilinum* var. *latiusculum* (Desv.) Underw. ex A. Heller | Dennstaedtiaceae | da1; da1-gu1; dai1-ga1; da1-li1 | QTB-JL-10 | leaves | food: cooked vegetable (86) | 86 | 0.683 |
| *Elaeagnus umbellata* Thunb. | Elaeagnaceae | ra1-lu1 | QTB-JL-18 | fruits | food: fruit (43), raw. | 43 | 0.341 |
| *Hippophae salicifolia* D.Don | Elaeagnaceae | da1-ru1 | QTB-JL-16 | fruits; branches | food: fruit (21), raw; seasoning (14), fruit juice is used as a substitute for vinegar; medicine: arthrophlogosis (3), the juice is used to smear the joints; fuelwood (1), used to burn. | 39 | 0.31 |
| *Rhododendron anthopogon* D. Don | Ericaceae | po1-lu1 | QTB-JL-115 | branches; flowers | ritual use (91), used to burn in incense burner; fuelwood (1), used to burn; medicine: eyesache (4), arthrophlogosis (4), flowers are used to soak water; beverage (6), soak in water; economic (4), be sold in store. | 110 | 0.873 |
| *Rhododendron arboreum* Sm. | Ericaceae | mei3-duo1 | QTB-JL-30 | branches; stems | fuelwood (7), used to burn; craft (9), used to make Tibetan traditional wooden bowls. | 16 | 0.127 |
| *Rhododendron lepidotum* Wall. ex G. Don | Ericaceae | su1-lu1 | QTB-JL-114 | branches | ritual use (15), used to burn incense burner | 15 | 0.119 |
| *Euphorbia micractina* Boiss. | Euphorbiaceae | ta3-lu1-ma1 | QTB-JL-85 | leaves | medicine: poisons (2) | 2 | 0.016 |
| *Cicer microphyllum* Benth. | Fabaceae | pu3-gui3 | EBT-PL-13 | fruits | food: fruit (2), raw. | 2 | 0.016 |
| *Quercus semecarpifolia* Sm. | Fagaceae | bai1-luo4 | QTB-JL-25 | stems; branches; leaves | ritual use (2), used to burn in incense burner; craft (15), used to make Tibetan traditional wooden bowls; fuelwood (48); fodder (5), used to feed cattle; food: starch (4), cooked fruit. | 74 | 0.587 |
| Gentiana veitchiorum Hemsl. | Gentianaceae | bang1-jie1-mei3-duo3 | QTP-EBT-3024 | whole plant | medicine: fever (18), soaked in water. | 18 | 0.143 |
| *Swertia cordata* (Wall. ex G. Don) C.B. Clarke | Gentianaceae | di1-ge1-da1 | QTP-EBT-3111 | aerial parts | medicine: fever (10), soak in water; economic (2); veterinary medicine (1), soak in water. | 13 | 0.103 |
| *Isoetes hypsophila* Hand.-Mazz. | Isoetaceae | pa1-xia4 | QTP-JPG-3 | leaves | food: vegetable (25), cooked vegetable. | 25 | 0.198 |
| *Juglans regia* L. | Juglandaceae | da1-ba1 | QTB-JL-88 | fruits; stems; branches | dyes (23), pericarp used to dye the container black; ritual use (9), used to burn in incense burner; food: fruit (9), raw; craft (30), used to make Tibetan traditional wooden bowls; fuelwood (12), used to burn. | 83 | 0.659 |
| *Elsholtzia fruticosa* (D.Don) Rehder | Lamiaceae | ma1-zei1 | QTB-JL-48 | aerial parts | ritual use (12), used to burn in incense burner; fuelwood (5), used to burn. | 17 | 0.135 |
| *Nepeta densiflora* Kar. & Kir. | Lamiaceae | pi1-ba4 | QTP-EBT-3060 | aerial parts | fodder (3), used to feed cattle | 3 | 0.024 |
| *Fritillaria cirrhosa* D.Don | Liliaceae | bai1-mu4 | QTP-EBT-3012 | bulbs | medicine: tonic (40), stew or soak in water; cold (20); economic (45), be sold in store; veterinary medicine (1), soak in water; food: fruit (2), raw. | 108 | 0.857 |
| *Malva verticillata* L. | Malvaceae | jiang4-ba1-la1-mu1 | QTB-JL-36 | roots; leaves | food: vegetable (25), cooked vegetable. | 25 | 0.198 |
| *Paris polyphylla* Sm. | Melanthiaceae | bo1-luo3 | QTP-EBT-3085 | arieal parts | ritual use (11), used to burn in incense burner; medicine: stomachache, soaked in water or wine (10); economic (2), be sold in store; vegetable (28), cooked vegetable. | 51 | 0.405 |
| *Gastrodia elata* Blume | Orchidaceae | tian3-ma3 | QTP-JPG-3292 | roots | economic (29), be sold in store; medicine: headache (6), cardiopathy (40), slice and soak in water or stew; food: vegetable (2), used to make soup with chicken. | 77 | 0.611 |
| *Gymnadenia orchidis* Lindl. | Orchidaceae | wang1-bu1-la1-ba1 | QTB-JL-56 | roots | medicine: pulmonary disease (5), soak in water or wine; burn (32), acne (38), used to daub affected area; economic (31); ritual use (10), raw materials for making Tibetan incense. | 116 | 0.921 |
| *Phytolacca acinosa* Roxb. | Phytolaccaceae | wo1-yang1 | QTB-JL-84 | leaves | food: vegetable (13), cooked vegetable. | 13 | 0.103 |
| *Larix potaninii* var. *himalaica* (W.C.Cheng & L.K.Fu) Farjon & Silba | Pinaceae | long3-xin1 | QTP-JPG-7 | branches; stems | fuelwood (4), used to burn; craft (9), used to make Tibetan traditional wooden bowls. | 13 | 0.103 |
| *Pinus wallichiana* A.B.Jacks. | Pinaceae | nong1-xin1; tang3-xin1 | QTB-JL-39 | branches; stems | fuelwood (44), used to burn; craft (7), used to make Tibetan traditional wooden bowls; ritual use (11), used to burn in incense burner; food: vegetable (7), cooked vegetable. | 73 | 0.579 |
| *Neopicrorhiza scrophulariiflora* (Pennell) D.Y.Hong | Plantaginaceae | di1-da1; hong1-lei1 | QTB-JL-67 | roots | medicine: cold and fever (107), soaked in water; economic (18), be sold in store; veterinary medicine: fever (3), soak in water. | 128 | 1.016 |
| *Plantago asiatica* L. | Plantaginaceae | wo1-ma1-ka1 | QTP-EBT-3117 | aerial parts | medicine: hypertension (4), soaked in water; food: vegetable (3), cooked vegetable. | 7 | 0.056 |
| *Plantago asiatica* subsp. *densiflora* (J.Z.Liu) Z.Y.Li | Plantaginaceae | ou3-ma1-ka3 | QTB-JL-12 | leaves; roots | medicine: hypertension (2), soaked in water; food: vegetable (3), cooked vegetable. | 5 | 0.04 |
| *Avena fatua* L. | Poaceae | sei1-za1-ba1 | QTB-JPG-11 | aerial parts | fodder (1), used to feed cattle. | 1 | 0.008 |
| *Fargesia* sp. | Poaceae | niu1-dong1 | QTB-JL-118 | stems | economic (1), be sold in store; food: vegetable (66), cooked vegetable; craft (19), used to make bamboo plaits; ritual use (6), used to burn in incense burner; fuelwood (2), used to burn; fodder (7), used to feed cattle. | 101 | 0.802 |
| *Poaceae* sp. | Poaceae | zang4-ong1-bu4 | QTP-JPG-8 | whole plant | fodder (7), used to feed cattle. | 7 | 0.056 |
| *Fagopyrum esculentum* Moench | Polygonaceae | bai1-bi1-ya1 | QTB-JL-60 | arieal parts | fodder (3), used to feed cattle | 3 | 0.024 |
| *Fallopia denticulata* (C.C.Huang) Holub | Polygonaceae | a1-lang1-ba1-lang1 | QTB-JL-122 | aerial parts; roots | fodder (2), used to feed cattle; medicine: diarrhea (5), hair follicle (2), soak in water. | 9 | 0.071 |
| *Koenigia tortuosa* (D.Don) T.M.Schust. & Reveal | Polygonaceae | nia1-luo1 | QTB-JL-4 | aerial parts; stems | dye (6), used to dye wooden bowls or clothes yellow; fodder (8), used to feed cattle; food: fruit (4), raw. | 18 | 0.143 |
| Pteroxygonum denticulatum (C.C.Huang) T.M.Schust. & Reveal | Polygonaceae | ren3-bu1 | QTB-JL-45 | aerial parts | fodder (2), used to feed cattle. | 2 | 0.016 |
| *Rheum australe* D. Don | Polygonaceae | qu1-wa1; jiong1 | QTB-JL-3 | stems; roots | fruit (17), raw eat tender stem; dye (53), used to dye wooden bowls or clothes yellow. | 70 | 0.556 |
| *Rumex nepalensis* Spreng. | Polygonaceae | xiu1-ma1 | EBT-PL-86 | aerial parts | fodder (2), used to feed cattle. | 2 | 0.016 |
| *Aconitum jilongense* W.T.Wang & L.Q.Li | Ranunculaceae | beng3-ga1 | QTB-JPG-1 | roots | medicine: diarrhea (24), soak in water. | 24 | 0.19 |
| *Clematis rehderiana* Craib | Ranunculaceae | ba1-ji1-ma1 | EBT-PL-84 | leaves | food: vegetable (2), cooked vegetable. | 2 | 0.016 |
| *Delphinium kamaonense* Huth | Ranunculaceae | jia1-bei1-mei1-duo1 | QTB-JL-37 | aerial parts | fodder (1), used to feed cattle. | 1 | 0.008 |
| *Eriocapitella rivularis* (Buch.-Ham. ex DC.) Christenh. & Byng | Ranunculaceae | cei1-di1-ma1 | QTB-JPG-9 | aerial parts | fodder (1), used to feed cattle. | 1 | 0.008 |
| *Gymnaconitum gymnandrum* (Maxim.) Wei Wang & Z.D.Chen | Ranunculaceae | zen1-du1; du3-wa1-ten3-du1 | QTP-EBT-3097 | roots | medicine: poisons (16), rheumatism (16), soaked in water and apply to the affected area; economic (4), be sold in store. | 36 | 0.286 |
| *Berchemia flavescens* (Wall.) Wall. ex Brongn. | Rhamnaceae | bo1-ge1-da4 | QTB-JL-93 | fruits | food: fruit (45), raw. | 45 | 0.357 |
| *Argentina anserina* (L.) Rydb. | Rosaceae | chu1-ma1 | QTP-EBT-3055 | roots | food: starche (60), cooked and eat with yogurt or rice. | 60 | 0.476 |
| *Chaenomeles thibetica* T.T.Yu | Rosaceae | bai1-la1 | QTB-JL-109 | fruits | food: fruit (19), raw; fuelwood (3), used to burn. | 22 | 0.175 |
| *Fragaria nubicola* (Lindl. ex Hook.f.) Lacaita | Rosaceae | long1-mei1; sei1-duo1-zhe3-xin1 | QTB-JL-9 | fruits; stems | fruit (83), raw; ritual use (3), used to burn in incense burner. | 86 | 0.683 |
| *Griffitharia vestita* (Wall. ex G.Don) Rushforth | Rosaceae | na1-zi1 | QTB-JL-5 | fruits; branches | food: fruit (55), raw; fuelwood (1), used to burn; ritual use (2), used to burn in incense burner. | 58 | 0.46 |
| *Prinsepia utilis* Royle | Rosaceae | bu1-long1-che4-mang1 | QTB-JL-38 | seeds | economic (9), be sold in store. | 9 | 0.071 |
| *Prunus holosericea* (Batal.) Kost. | Rosaceae | a1-lu1-ba3-lu1 | QTB-JL-91 | fruits | food: fruit (6), raw. | 6 | 0.048 |
| *Prunus mira* Koehne | Rosaceae | kang3-bu4 | QTB-JL-69 | fruits | food: fruit (53), raw. | 53 | 0.421 |
| *Rosa macrophylla* Lindl. | Rosaceae | sei1-duo1 | QTB-JL-29 | branches; fruits | fuelwood (3), used to burn; food: fruit (11), raw. | 14 | 0.111 |
| *Rosa sericea Lindl.* | Rosaceae | gu1-jiu1-ma1; gun1-zhong1 | QTB-JL-17 | fruits; branches | food: fruit (91), raw; fuelwood (1), used to burn; medicine: digestion (1), raw. | 94 | 0.746 |
| *Rubus aurantiacus* Focke ex Sarg. | Rosaceae | ni1-na1 | QTB-JL-14 | fruits | food: fruit (6), raw. | 6 | 0.048 |
| *Rubus austrotibetanus* T.T.Yu & L.T.Lu | Rosaceae | nia1-lang1 | QTB-JL-82 | fruits | food: fruit (55), raw. | 55 | 0.437 |
| *Rubus biflorus* Buch.-Ham. ex Sm. | Rosaceae | nie1-sen1; nia1-lang1 | QTB-JL-83 | fruits | food: fruit (9), raw. | 9 | 0.071 |
| *Rubus niveus* Thunb. | Rosaceae | nia1-lang2 | QTB-JL-13 | fruits | food: fruit (68), raw. | 68 | 0.54 |
| *Thomsonaria ochracea* (Hand.-Mazz.) Rushforth | Rosaceae | ca1-le1-ba1 | QTB-JL-92 | branches | fuelwood (17), used to burn; tool (3), used to make handle; ritual use (1), used to burn in incense burner. | 21 | 0.167 |
| *Zanthoxylum bungeanum* Maxim. | Rutaceae | ei1-ma1 | QTB-JL-8 | fruits; seeds | food: seasoning (83), vegetable (14), cooked it with meat; economic (3), be sold in store; medicine: endocrine (3), raw or soak in water; fuelwood (1), used to burn. | 104 | 0.825 |
| *Salix babylonica* f. *babylonica* | Salicaceae | jiang1-ma1 | QTB-JL-108 | branches | fodder (5), used to feed cattle; fuelwood (3), used to burn; ritual use (3), used to burn in incense burner. | 11 | 0.087 |
| *Salix trichocarpa* C.F. Fang | Salicaceae | lang1-ma1 | QTB-JL-47 | branches; flowers; stems | fuelwood (15), used to burn; ritual use (14), used to burn in incense burner; fodder (1), used to feed cattle; craft (3), used to make wooden bowl; food: vegetable (2), flower buds can be fried and eaten. | 35 | 0.278 |
| *Schisandra elongata* (Blume) Baill. | Schisandraceae | gong1-zhu1 | QTB-JL-117 | fruits | food: fruit (7), raw. | 7 | 0.056 |
| *Tamarix chinensis* Lour. | Tamaricaceae | ong1-bu4 | QTB-JL-18 | branches | ritual use (1), burned to sacrifice to the dead. | 1 | 0.008 |
| *Taxus wallichiana* Zucc. | Taxaceae | sei1-ge1-xia4 | QTB-JL-31 | branches; fruits | fuelwood (11), used to burn; food: fruit (5), raw. | 16 | 0.127 |
| *Urtica ardens* Link | Urticaceae | suo3-wa1 | QTP-JPG-5 | leaves | food: vegetable (36), cooked vegetable. | 36 | 0.286 |
| *Urtica urens* L. | Urticaceae | suo3-wa1 | QTP-JPG-4 | leaves | food: vegetable (23), cooked vegetable. | 23 | 0.183 |
| *Viburnum cotinifolium* D. Don | Viburnaceae | gei1-jiu1-ma1 | QTB-JL-51 | fruits | food: fruit (4), raw. | 4 | 0.032 |
| *Viburnum nervosum* D. Don | Viburnaceae | ka3-la1-suo1 | QTB-JL-102 | fruits | food: fruit (6), raw. | 6 | 0.048 |
